# Disease-associated and patient-specific immune cell signatures in juvenile-onset systemic lupus erythematosus: patient stratification using a machine-learning approach

**DOI:** 10.1016/S2665-9913(20)30168-5

**Published:** 2020-07-29

**Authors:** George A Robinson, Junjie Peng, Pierre Dönnes, Leda Coelewij, Meena Naja, Anna Radziszewska, Chris Wincup, Hannah Peckham, David A Isenberg, Yiannis Ioannou, Ines Pineda-Torra, Coziana Ciurtin, Elizabeth C Jury

**Affiliations:** a Centre for Rheumatology Research, Department of Medicine, University College London, London, UK; b Centre for Adolescent Rheumatology Versus Arthritis, Department of Medicine, University College London, London, UK; c Centre for Cardiometabolic and Vascular Science, Department of Medicine, University College London, London, UK; d SciCross AB, Skövde, Sweden; e UCB Pharma, Immunology Translational Medicine, Slough, UK

## Abstract

**Background:**

Juvenile-onset systemic lupus erythematosus (SLE) is a rare autoimmune rheumatic disease characterised by more severe disease manifestations, earlier damage accrual, and higher mortality than in adult-onset SLE. We aimed to use machine-learning approaches to characterise the immune cell profile of patients with juvenile-onset SLE and investigate links with the disease trajectory over time.

**Methods:**

This study included patients who attended the University College London Hospital (London, UK) adolescent rheumatology service, had juvenile-onset SLE according to the 1997 American College of Rheumatology revised classification criteria for lupus or the 2012 Systemic Lupus International Collaborating Clinics criteria, and were diagnosed before 18 years of age. Blood donated by healthy age-matched and sex-matched volunteers who were taking part in educational events in the Centre for Adolescent Rheumatology Versus Arthritis at University College London (London, UK) was used as a control. Immunophenotyping profiles (28 immune cell subsets) of peripheral blood mononuclear cells from patients with juvenile-onset SLE and healthy controls were determined by flow cytometry. We used balanced random forest (BRF) and sparse partial least squares-discriminant analysis (sPLS-DA) to assess classification and parameter selection, and validation was by ten-fold cross-validation. We used logistic regression to test the association between immune phenotypes and k-means clustering to determine patient stratification. Retrospective longitudinal clinical data, including disease activity and medication, were related to the immunological features identified.

**Findings:**

Between Sept 5, 2012, and March 7, 2018, peripheral blood was collected from 67 patients with juvenile-onset SLE and 39 healthy controls. The median age was 19 years (IQR 13–25) for patients with juvenile-onset SLE and 18 years (16–25) for healthy controls. The BRF model discriminated patients with juvenile-onset SLE from healthy controls with 90·9% prediction accuracy. The top-ranked immunological features from the BRF model were confirmed using sPLS-DA and logistic regression, and included total CD4, total CD8, CD8 effector memory, and CD8 naive T cells, Bm1, and unswitched memory B cells, total CD14 monocytes, and invariant natural killer T cells. Using these markers patients were clustered into four distinct groups. Notably, CD8 T-cell subsets were important in driving patient stratification, whereas B-cell markers were similarly expressed across the cohort of patients with juvenile-onset SLE. Patients with juvenile-onset SLE and elevated CD8 effector memory T-cell frequencies had more persistently active disease over time, as assessed by the SLE disease activity index 2000, and this was associated with increased treatment with mycophenolate mofetil and an increased prevalence of lupus nephritis. Finally, network analysis confirmed the strong association between immune phenotype and differential clinical features.

**Interpretation:**

Machine-learning models can define potential disease-associated and patient-specific immune characteristics in rare disease patient populations. Immunological association studies are warranted to develop data-driven personalised medicine approaches for treatment of patients with juvenile-onset SLE.

**Funding:**

Lupus UK, The Rosetrees Trust, Versus Arthritis, and UK National Institute for Health Research University College London Hospital Biomedical Research Centre.

## Introduction

Systemic lupus erythematosus (SLE) is a chronic, multisystem autoimmune rheumatic disease with a complex aetiology.^1^ Juvenile-onset SLE accounts for approximately 15–20% of all cases and is defined by disease onset in childhood or adolescence (diagnosis before the age of 18 years).^2^ Juvenile-onset SLE has a more aggressive disease presentation than does adult-onset SLE. The juvenile-onset form is characterised by increased renal and CNS involvement and more severe haematological manifestations as well as a notable increase in cardiovascular disease risk compared with the adult-onset form.^3^, ^4^, ^5^ The heterogeneity of juvenile-onset SLE clinical manifestations is matched by a broad range of genetic and immunological abnormalities.^2^ No juvenile-onset SLE-specific medications are available, due mainly to the paucity of clinical trial data in children and adolescents, meaning that patients with juvenile-onset SLE are treated similarly to patients with adult-onset SLE.^2^, ^4^, ^6^, ^7^ However, despite treatment, severe juvenile-onset SLE leads to early organ damage and unsatisfactory outcomes (eg, renal and CNS manifestations) for many patients, emphasising the need for improved understanding of the immunological defects driving disease pathogenesis and clinically relevant patient stratification strategies for personalised treatment.

**Research in context**

**Evidence before this study**

Juvenile-onset systemic lupus erythematosus (SLE) is a rare autoimmune rheumatic disease characterised by a broad array of clinical manifestations associated with multiple genetic and immunological abnormalities; the condition has a more aggressive disease presentation than adult-onset SLE, emphasising the need for improved understanding of the immunological defects driving disease pathogenesis. We searched PubMed, Web of Science, and Google Scholar for research articles published between Jan 1, 1990, and March 1, 2020, using search terms including “(juvenile-onset) systemic lupus erythematosus”, “machine learning”, “immune signatures”, and “stratification”. We also searched for research articles published in the same time window in rheumatology-specific journals. Published abstracts were excluded from the searches. The earliest referenced article was published in 1993; however, due to the modern computational analytical techniques used in this paper, the majority of articles referenced were more recent (since 2016). We found that in-depth computational analysis of multi-omic datasets has accelerated the understanding of complex heterogeneous diseases such as SLE and juvenile-onset SLE. We also found some studies using machine-learning strategies to explore longitudinal gene expression signatures in peripheral blood from patients with juvenile-onset SLE that have identified unique patient groups.

**Added value of this study**

This study is the first to report an in-depth analysis of immune cell phenotype in patients with juvenile-onset SLE. Machine-learning methods identified a juvenile-onset SLE immune cell signature that stratified patients according to their disease trajectory.

**Implications of all the available evidence**

The application of machine-learning approaches to immune cell phenotyping data has identified immunological biomarkers that could help to unravel underlying disease mechanisms in juvenile-onset SLE and explain the differences in long-term outcomes of patients with juvenile-onset SLE. Such immunological signatures could facilitate better stratification of patients for optimal treatment choices and provide information to improve interventional clinical trial design.

Machine learning is a subdivision of artificial intelligence that builds analytical models through learning by example and has been used in a wide range of clinical areas, including pharmaceutical target prediction for drug discovery^8^ and disease diagnosis and prognosis.^9^ It relies on data collection and preparation, model training and evaluation, and multiple performance cycles for self-improvement, resulting in increased predictive power.

In the past 4 years, in-depth computational analysis of multi-omic datasets has accelerated the understanding of complex heterogeneous diseases such as SLE and juvenile-onset SLE.^10^, ^11^, ^12^, ^13^ A retrospective study of previous longitudinal gene expression data from paediatric and adult SLE populations identified three stratified groups within each cohort with unique disease activity and trajectories, supporting strategies to identify clinically informative groups using immune profiling.^13^

Another study applied three different machine-learning approaches, including k-nearest neighbours, generalised logistic models, and random forest models to predict disease activity in patients with SLE using whole-genome gene expression profiles.^14^ The random forest classifier outmatched other approaches by achieving 83% accuracy under ten-fold cross-validation.^14^ Another study used random forest models to predict lupus nephritis outcomes.^15^ We aimed to apply machine-learning approaches to immune cell frequency profiles, and clinical and serological data from patients with juvenile-onset SLE, to identify predictive disease outcome signatures.

## Methods

### Study design and participants

In this study, peripheral blood was collected from patients who attended the University College London Hospital (London, UK) adolescent rheumatology service. Patients were eligible to be included in the study if they had juvenile-onset SLE according to the American College of Rheumatology revised classification criteria for lupus (1997) or the Systemic Lupus International Collaborating Clinics (SLICC) criteria (2012), and were diagnosed before the age of 18 years. Full details of inclusion and exclusion criteria and relevant protocol details are in the appendix (p 1). Patient and disease characteristics (including demographics, age at onset, disease duration, clinical and serological parameters, and medication) were collected retrospectively from medical records and through questionnaires at the time of blood sampling. Disease activity was calculated using the SLE disease activity index 2000 (SLEDAI-2K). A score of 4 or more was used to indicate active disease. Lupus low disease activity state values were recorded for all patients.^16^ Disease parameters and treatment at subsequent clinical appointments were also collected longitudinally from baseline (time of immune phenotype analysis) to the most recent clinical appointment. Blood samples donated by healthy age-matched volunteers taking part in educational events in the Centre for Adolescent Rheumatology Versus Arthritis at University College London (London, UK) were used as controls. All participants had established puberty (Tanner stage 4–5). All information was stored as pseudo-anonymised data.

This study was approved by the London-Harrow Research Ethics Committee, reference 11/LO/0330. Written informed consent was acquired from patients and healthy controls.

### Multiparameter flow cytometry

Peripheral blood mononuclear cells (PBMCs; 1 × 10^6^) were stained with fixable blue dead cell stain (ThermoFisher, Carlsbad, CA, USA) and a T-cell or antigen presenting cell antibody panel followed by subsequent washes and fixation in 2% paraformaldehyde. Data acquisition was on a BD LSRFORTESSA X-20 flow cytometer (BD Biosciences, San Jose, CA, USA; 1 × 10^6^–2 × 10^6^ cells per sample), and FlowJo Analysis Software (TreeStar, San Jose, CA, USA) was used to assess frequencies of 28 immune cell subsets. Cytometer Setup and Tracking (BD Biosciences) beads were run to assess cytometer performance. Application settings were created and applied to panel templates before fluorochrome compensation to ensure that all immunophenotyping data were comparable over time. The list of antibodies used, markers used to identify cell types by flow cytometry, and gating strategy are included in the appendix (pp 2–4).

### Statistical analysis

Group averages over time or spaghetti plots for individual patient trajectories were analysed. We compared immunophenotype data between healthy controls and patients with juvenile-onset SLE using an unpaired *t* test or across stratified groups of patients with juvenile-onset SLE using one-way ANOVA. Data were corrected for multiple testing using a false discovery rate of 5%. We used supervised machine-learning approaches—balanced random forest (BRF) and sparse partial least squares-discriminant analysis (sPLS-DA)—for classification and parameter selection. Because juvenile-onset SLE is a rare disease, we used a BRF machine-learning approach^14^, ^15^ to further define and validate the juvenile-onset SLE immune cell profile (see the appendix pp 5–8 for the description of machine-learning approach). This approach can overcome difficulties in obtaining validation datasets because the model does not overfit to training data. Validation was by ten-fold cross-validation. We used logistic regression to assess the association between immunophenotypes (28 parameters) and juvenile-onset SLE. Important parameters identified by all analysis approaches were selected and used for patient stratification by k-means clustering; for this analysis, immunological parameters fulfilling the following criteria were selected: top ten important variables in BRF model; top ten weighting variables in sPLS-DA analysis; and parameters significantly associated with juvenile-onset SLE in logistic regression analysis. Clinical trajectory analysis was used to identify the clinical difference between patient groups. Demographic variables, including sex, age, and ethnicity, were adjusted for as appropriate. ANOVA was done in GraphPad Prism 8 software, and other statistical analyses was done in R (version 3.5.2). For detailed description of the analysis and software packages used see the appendix (p 8).

### Role of the funding source

The funders of the study had no role in study design, data collection, data analysis, data interpretation, or writing of the report. The corresponding author had full access to all the data in the study and had final responsibility for the decision to submit for publication.

## Results

**Figure 1:**
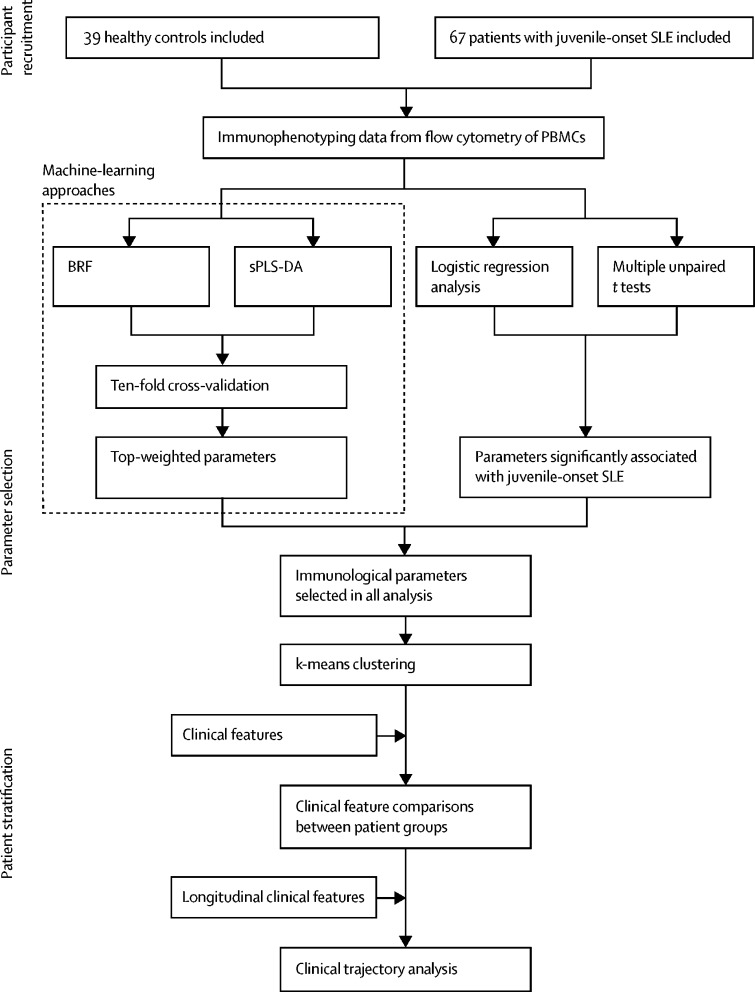
Study design and analysis plan flow diagram BRF=balanced random forest. PBMCs=peripheral blood mononuclear cells. SLE=systemic lupus erythematosus. sPLS-DA=sparse partial least squares-discriminant analysis.

Between Sept 5, 2012, and March 7, 2018, peripheral blood from 67 patients with juvenile-onset SLE and 39 healthy controls was collected (figure 1). The median age for both populations was similar (19 years [IQR 13–25] *vs* 18 years [16–25]; table). Fewer males were recruited in the juvenile-onset SLE group, reflecting female bias of the disease. The healthy control group had fewer Asian and black participants; these demographic differences were adjusted for in subsequent analyses. Patient follow-up was between Sept 5, 2012, and Dec 23, 2019 (mean duration 4·9 years [SD 1·4], mean number of visits per patient 17·1 [7·8]).

**Table:** Demographic and clinical table of all patients and healthy controls

		**Healthy controls (n=39)**	**Patients with juvenile-onset SLE (n=67)**
Sex			
	Female	22 (56%)	54 (81%)
	Male	17 (44%)	13 (19%)
Age, years		18 (16–25)	19 (13–25)
Body-mass index, kg/m^2^		23·1 (19·9–24·5)	22·41 (20·3–26·6)
Ethnicity			
	White	20 (51%)	20 (30%)
	Asian	10 (26%)	24 (36%)
	Black	2 (5%)	17 (25%)
	Other or unknown	7 (18%)	6 (9%)
Disease characteristics			
	Age at diagnosis	..	12·2 (6·4)
	Disease duration, years	..	7·1 (4·9)
	SLEDAI-2K	..	2·4 (0·0–4·0)
	SLEDAI-2K ≥4	..	10 (15%)
	SLEDAI-2K <4	..	57 (85%)
	Systemic Lupus International Collaborating Clinics	..	0·1 (0·4)
	Lupus low disease activity state	..	49 (73%)
Current organ involvement			
	Neurological	..	12 (18%)
	Serositis	..	9 (13%)
	Cutaneous	..	57 (85%)
	Haematological	..	28 (42%)
	Musculoskeletal	..	55 (82%)
	Renal	..	21 (31%)
Serology			
	Anti-dsDNA antibodies, IU/mL (normal range ≤50)	..	14 (2–154)
	Anti-dsDNA antibodies outside normal range	..	24 (36%)
	CRP, mg/L (normal range <5)	..	1·00 (0·60–2·55)
	CRP, outside normal range	..	8 (12%)
	Complement component C3, g/L (normal range 0·9–1·8)	..	1·02 (0·76–1·21)
	Complement component C3 outside normal range	..	24 (36%)
	Lymphocyte count, ×10^9^ cells per L (normal range 1·3–3·5)	..	1·50 (1·28–2·06)
	Lymphocyte count outside normal range	..	34 (51%)
	Neutrophil count, ×10^9^ cells per L (normal range 2·0–7·5)	..	3·01 (2·22–7·68)
	Neutrophil count outside normal range	..	19 (28%)
	Urine protein:creatinine ratio, mg/mmol (normal range 0–13)	..	8 (6–13)
	Urine protein:creatinine ratio outside normal range	..	16 (24%)
	Haemoglobin, g/L (normal range 115–155)	..	122 (112–134)
	Haemoglobin outside normal range	..	25 (37%)
	Platelet count, ×10^9^ cells per L (normal range 150–400)	..	276 (211–337)
	Platelet count outside normal range	..	7 (10%)
	Antinuclear antibody positive	..	55 (82%)
	Extractable nuclear antigen-positive	..	42 (63%)
Clinical lipids			
	Cholesterol, mmol/L (normal range <5)	..	4·0 (3·4–4·3)
	Triglycerides, mmol/L (normal range <3)	..	0·8 (0·6–1·15)
	HDL cholesterol, mmol/L (normal range >1)	..	1·5 (1·2–1·7)
	LDL cholesterol, mmol/L (normal range <3)	..	2·1 (1·6–2·4)
	Cholesterol:HDL cholesterol ratio (normal range <4)	..	2·75 (2·30–3·28)
Current treatment			
	Hydroxychloroquine	..	62 (93%)
	Mycophenolate mofetil	..	27 (40%)
	Prednisolone	..	32 (48%)
	Vitamin D	..	13 (19%)
	Methotrexate	..	6 (9%)
	Azathioprine	..	15 (22%)

Data are n (%), median (IQR), or mean (SD). CRP=C-reactive protein. SLE=systemic lupus erythematosus. SLEDAI-2K=SLE disease activity index 2000.

**Figure 2:**
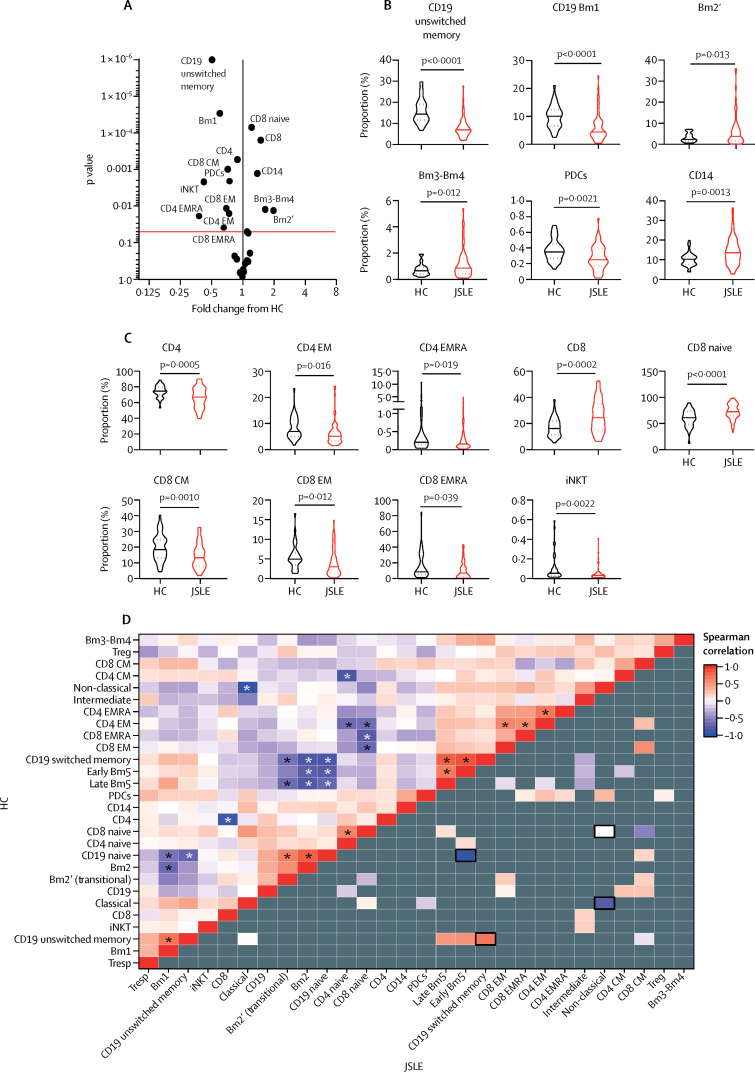
The immunological architecture is altered in juvenile-onset SLE (A) Volcano plot displaying comparison between patients with juvenile-onset SLE and heath controls. Fold change versus log_10_ p values are displayed from unpaired *t* tests. The red line indicates adjusted p value following 5% false discovery rate adjustment for multiple comparisons. (B, C) Violin plots displaying antigen presenting cells (panel B) and T-cell subsets (panel C) that were significantly different between healthy controls and patients with juvenile-onset SLE by unpaired *t* test. The solid line indicates the mean and the dashed line indicates the SE. Adjusted p values are shown. (D) Correlation comparison analysis performed on immune phenotyping data described in panel A. The upper left of the heat map shows the correlation between immune cell types (28 immunological variables) in healthy controls. Spearman correlation coefficients for each pair of cell types are represented by colour. Asterisks indicate significant correlations, p<0·05. The bottom right of the heat map shows the correlation between immune cell types in patients with juvenile-onset SLE. Grey indicates that the Spearman correlation coefficient is not signficantly different from that of healthy controls. Significantly different correlations in patients with juvenile-onset SLE compared with healthy controls are coloured (p<0·05) and outlined in black (p<0·01). CM=central memory. EM=effector memory. EMRA=effector memory cells re-expressing CD45RA. HC=healthy controls. iNKT=invariant natural killer T cells. JSLE=juvenile-onset SLE. PDCs=plasmacytoid dendritic cells. SLE=systemic lupus erythematosus. Treg=regulatory T cells. Tresp=responder T cells.

In-depth immune cell phenotyping of PBMCs showed that patients with juvenile-onset SLE had a disrupted immune cell profile compared with healthy controls (figure 2A–C; see appendix p 4 for gating strategies), including defects in T-cell, B-cell, and monocyte populations. Specifically, these differences included an increase in total and naive CD8 T cells, total monocytes, and plasmablasts, as well a decrease in total CD4 T cells and memory T-cell and B-cell populations in patients with juvenile-onset SLE compared with healthy controls.

Correlation comparison analysis for immune cell profiles was conducted within healthy controls and within patients with juvenile-onset SLE to assess the relationships between immune cell subsets; significant negative correlations between naive and memory B-cell subpopulations and separately between naive and memory T-cell populations were identified in healthy controls (figure 2D, upper triangle; appendix p 9). However, in patients with juvenile-onset SLE, a clear global change in immunological architecture was evident compared with healthy controls. Many of the immune cell associations identified in healthy controls were inverted or exacerbated in patients with juvenile-onset SLE, and a significant disruption in the relationship between memory T-cell and B-cell subsets with each other, monocyte subsets, and plasmacytoid dendritic cells (PDCs) was evident (figure 2D, lower triangle; appendix p 9). These results show a comprehensive alteration of immune cell subsets with substantial memory lymphocyte involvement, indicating dysregulation of the adaptive immune system in juvenile-onset SLE.

**Figure 3:**
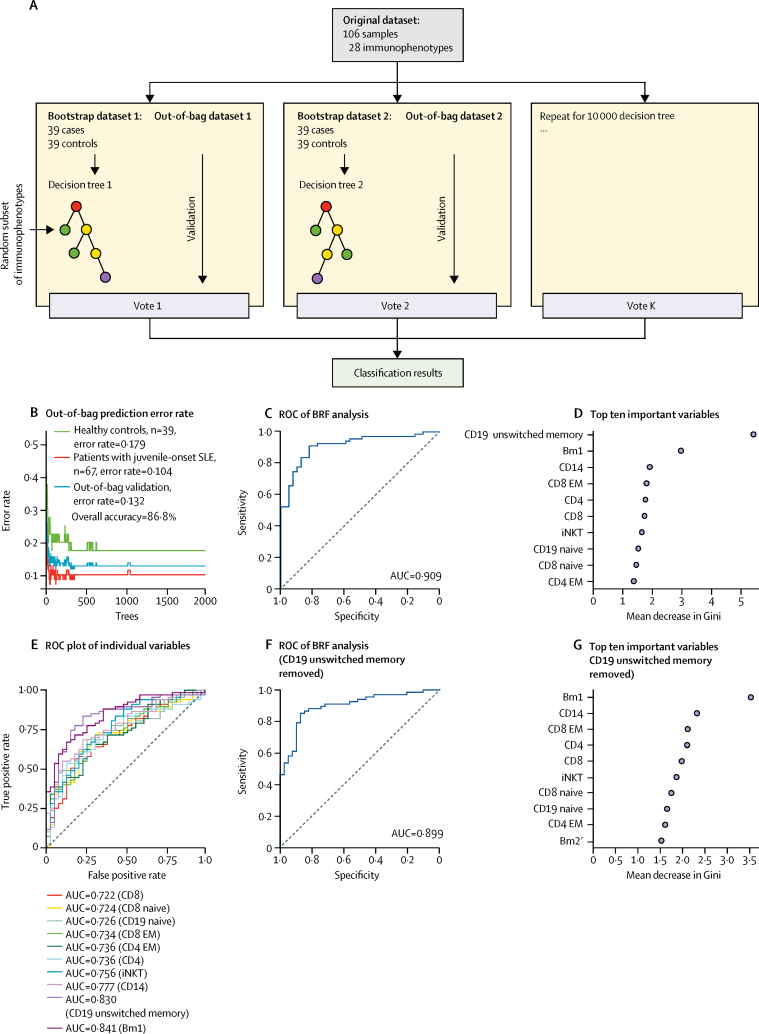
BRF analysis of immunophenotype data (A) Building a predictive model using a BRF approach (appendix pp 5–6). (B) Comparison of 28 different immune cell subsets in healthy controls (n=39) versus patients with juvenile-onset SLE (n=67) using the BRF model. (C) ROC analysis for the BRF model. (D) The top ten variables contributing to the BRF model are shown. The mean decrease in Gini measures the importance of each variable to the model: a higher score indicates a higher importance of the variable. (E) ROC with AUC from univariate models showing the sensitivity and specificty of the top ten markers identified by the model. (F) ROC analysis without CD19 unswitched memory B cells (the most predictive parameter). (G) The top ten contributing variables in the BRF model trained on 27 immunological parameters (excluding CD19+ unswitched memory cells). AUC=area under the curve. BRF=balanced random forest. EM=effector memory. iNKT=invariant natural killer T cells. ROC=receiver operating characteristic. SLE=systemic lupus erythematosus.

We used a BRF machine-learning approach to further define and validate the juvenile-onset SLE immune cell profile. After optimisation, the BRF model (figure 3A) distinguished patients with juvenile-onset SLE from healthy controls with a classification accuracy of 86·8% (figure 3B). The classification error rate in the out-of-bag validation set was 10·4% for predicting individuals with juvenile-onset SLE and 17·9% for predicting healthy controls. Receiver operating characteristic curve (ROC) analysis of the BRF model showed an area under the curve (AUC) of 0·909 (accuracy 90·9%; figure 3C), indicating outstanding model efficiency in discriminating patients with juvenile-onset SLE from healthy controls. From this analysis, the diagnostic sensitivity was 89·6% and specificity was 82·1%. In addition, the classification accuracy held a steady measure of 87·8% (sensitivity 89·6% and specificity 84·7%) in the ten-fold cross-validation analysis. Demographic variables (age, sex, and ethnicity) were also included in the model for adjustment purposes but did not appear in the top ten weighted variables. Thus, patients with juvenile-onset SLE were discriminated from healthy controls with high confidence using this BRF model generated with these immunological parameters.

The top contributing features segregating patients with juvenile-onset SLE from healthy controls were CD19 unswitched memory B cells, Bm1 (naive) B cells, and CD14 monocytes (figure 3D). However, individual random forest models using each of the top ten most important variables confirmed that each cell type played an important part in the original BRF model. The AUC of the univariate random forest models ranged from 0·722 to 0·841, with the best performance given by the Bm1-only model (AUC=0·841), followed by the CD19 unswitched memory B-cell-only model (AUC=0·830; figure 3E), showing that the multivariate BRF approach outperforms univariate models.

Furthermore, removing CD19 unswitched memory B cells (the top variable from the multivariate BRF model) did not substantially alter the predictive capacity of the model (AUC=0·899; figure 3F–G), suggesting that dysregulation of multiple immune cell subsets might better explain the complexity and heterogeneity of the disease phenotype.

**Figure 4:**
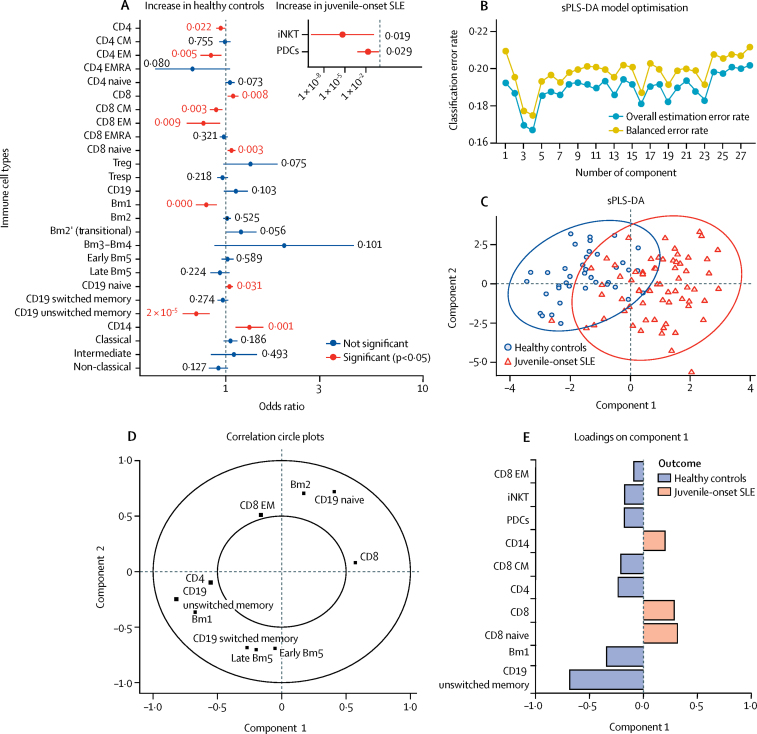
Top hits from BRF model validated with logistic regression analysis and sPLS-DA (A) Odds ratios (error bars indicate 95% CIs) of 28 immunological parameters were computed with univaraite logistic regression analysis. iNKT and PDC data is shown seperately inset because of very different CI values. (B) sPLS-DA model optimisation using ten-fold cross-validation. (C) sPLS-DA plot to validate the top hits from the predictive model. Individual distribution points and confidence ellipses (ovals) are plotted for the healthy control and juvenile-onset SLE groups. (D) Using this analysis, the weighting of each cell type in component 1 and 2 is displayed (inner circle is the 0·5 cutoff). (E) Factor loading weights in component 1 for the top ten ranked immunological parameters. The bars indicate the class with maximal mean value. Variables excluded from the plot have no weight in component 1. BRF=balanced random forest. CM=central memory. EM=effector memory. EMRA=effector memory cells re-expressing CD45RA. iNKT=invariant natural killer T cells. PDCs=plasmacytoid dendritic cells. SLE=systemic lupus erythematosus. sPLS-DA=sparse partial least squares-discriminant analysis. Treg=regulatory T cells. Tresp=responder T cells.

To validate the relationship between the individual immunological parameters and juvenile-onset SLE further, logistic regression analysis (adjusted for sex, ethnicity, and age) was applied by modelling the probability of juvenile-onset SLE using the immune profiles of the healthy control and juvenile-onset SLE cohorts (figure 4A; appendix p 10). 12 of 28 immune cell types were significantly associated with juvenile-onset SLE, substantiating the global immunological difference between juvenile-onset SLE and healthy controls. The correlation between having juvenile-onset SLE and the reduced frequency of CD19 unswitched memory B cells was relatively high (odds ratio 0·71 [95% CI 0·60–0·82]), in accordance with the BRF classification analysis (figure 2D). Indeed, all variables selected by the optimal BRF model were confirmed as significantly altered in patients with juvenile-onset SLE compared with healthy controls by logistic regression.

As a secondary validation, sPLS-DA was done to rank and validate the immunological variables by their distribution in patients with juvenile-onset SLE and healthy controls. sPLS-DA is a supervised clustering machine-learning approach that combines parameter selection and classification into one operation. By assessing the overall estimation error rate and balanced error rate in ten-fold cross-validation, models with four components were chosen for optimal model performance; these models gave the lowest overall estimation error rate (0·167) and balanced error rate (0·175; figure 4B). This analysis identified a significant separation between patients with juvenile-onset SLE and healthy controls by plotting principal component 2 against principal component 1 (figure 4C), indicating that principal component 1 predominantly separated the two groups and provided good prediction ability for the model. Similar to the BRF analysis, a subset of discriminating immune cell types were selected and ranked by discriminating capability (figure 4D–E). The highest weighted immunological parameter was CD19 unswitched memory B cells (–0·69), followed by Bm1 B cells (–0·34). The top ten discriminating parameters selected from sPLS-DA were all reported as significantly associated with juvenile-onset SLE and matched the most important parameters from the BRF model, with the exception of CD8 central memory T cells and PDCs. Thus, a distinct juvenile-onset SLE immune signature was identified and validated by different machine-learning methods that could discriminate patients with juvenile-onset SLE from healthy controls (appendix p 11).

**Figure 5:**
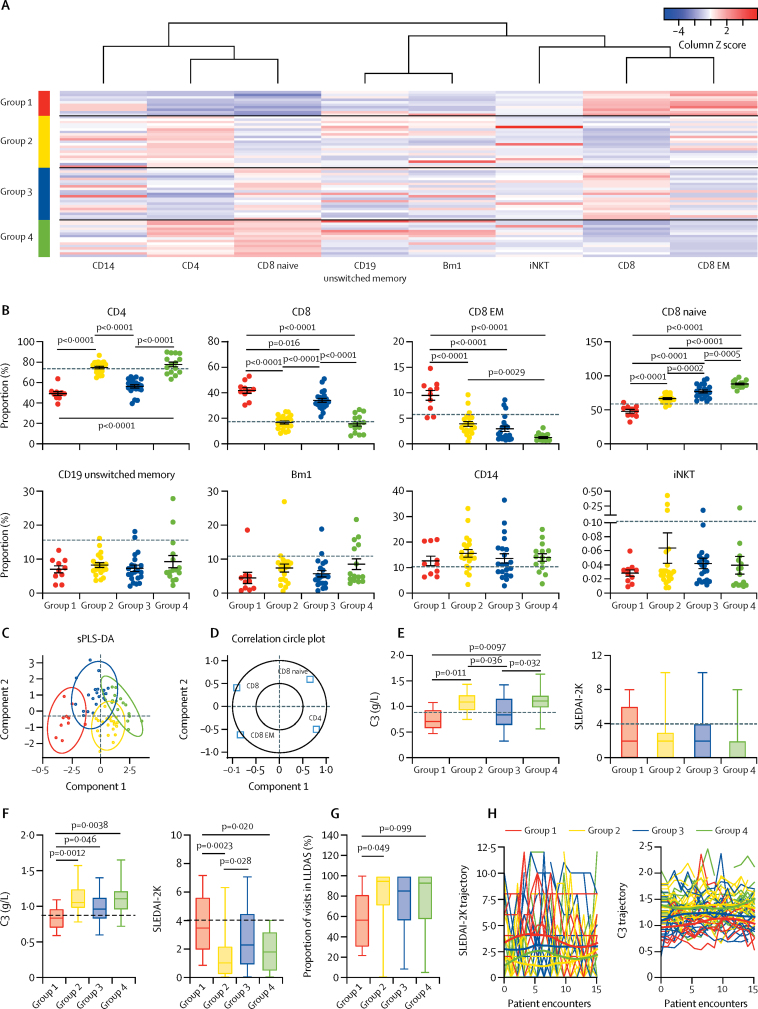
Patient clustering by top-weighted immunological parameters in patients with juvenile-onset SLE compared with healthy controls (A) Top-weighted immunological parameters of patients with juvenile-onset SLE (appendix pp 11–12) were stratified using k-means clustering. Immunophenotype is standardised within each column by Z score and plotted as a heat map, representing the relationship to the mean of the group (red represents relatively high frequency and blue represents relatively low frequency). Each row represents a patient with juvenile-onset SLE. Four groups of patients were recognised with distinct immune cell profiles. (B) Scatter dot plots displaying top-weighted immunological parameters between the k-means clustered groups. Mean (error bars indicate SE) was calculated with one-way ANOVA, and p values were calculated with Tukey's multiple comparisons test. The dashed lines represent the mean for the healthy control population for each cell type. (C) sPLS-DA plot showing the clustering of the validated top-weighted immunological parameters in patients with juvenile-onset SLE between k-means clustered juvenile-onset SLE groups. Individual distribution points and confidence ellipses (ovals) are plotted for each group. (D) Using this analysis, the weighting of each cell type in component 1 and 2 is displayed, where the inner circle is the 0·5 cutoff. (E) Box and whisker plots displaying baseline measures over 3–7 years of follow-up of clinical measures of disease activity between the k-means clustered groups of patients with juvenile-onset SLE. (F) Average measure over 3–7 years of follow-up of clinical measures of disease activity between the k-means clustered groups of patients with juvenile-onset SLE. Mean (error bars indicate SE) was calculated with one-way ANOVA, and p values were calculated with Tukey's multiple comparisons test. Dashed lines represent the clinical cutoff for active disease in the C3 plot and the assigned cutoff associated with active lupus in the SLEDAI-2K plot. (G) Box and whisker plot displaying longitudinal disease activity, using the same dataset from panel F, assessed as LLDAS. Mean (error bars indicate SE) was calculated with one-way ANOVA, and p values were calculated with Tukey's multiple comparisons test. (H) Individual patient trajectory of SLEDAI-2K and C3 over 15 clinical encounters displayed as spaghetti plots. Each line represents one patient with juvenile-onset SLE. Smoothing lines were added to indicate the trend of juvenile-onset SLE groups from previous k-means clustering. C3=complement component C3. iNKT=invariant natural killer T cells. LLDAS=lupus low disease activity state. SLE=systemic lupus erythematosus. SLEDAI-2K=systemic lupus erythematosus disease activity index 2000. sPLS-DA=sparse partial least squares-discriminant analysis.

To assess whether the juvenile-onset SLE signature could be used to stratify patients with juvenile-onset SLE further, k-means clustering, an unsupervised machine-learning algorithm was used. After screening, eight of 28 immune cell subsets were selected: total CD4, total CD8, CD8 effector memory (EM), CD8 naive, and invariant natural killer T cells; Bm1 and unswitched memory B cells; and total CD14 monocytes (figure 2B, C; appendix pp 11–12). Based on these variables, k-means clustering was done to stratify patients with juvenile-onset SLE into four groups (group 1, n=10; group 2, n=21; group 3, n=21; group 4, n=15; figure 5A).

Clear patterns among T-cell subsets were observed from the patient grouping, with significant differences in total CD4 and CD8, CD8 naive, and CD8 EM T-cell frequencies (figure 5B). Patients with juvenile-onset SLE in groups 1 and 3 shared elevated CD8 T-cell and reduced CD4 T-cell frequencies, compared with groups 2 and 4. Group 1 also had relatively high CD8 EM but low CD8 naive T cells. Notably, no significant differences were identified between the groups for unswitched memory and Bm1 B cells, CD14 monocytes, or invariant natural killer T cells. As a validation of the k-means clustered groups, sPLS-DA analysis revealed an excellent separation between the four groups; whereas principal component 1 provided some separation between the four groups, principal component 2 further discriminated between groups 3 and 4 and between groups 1 and 2 (figure 5C), which was driven by total, naive, and EM CD8 and total CD4 T cells (figure 5D). Of note, the activation status of T cells was not altered between the groups, whereas activation of B cells and monocytes, measured by HLA-DR expression, was significantly elevated in patients in group 1 (appendix p 13).

Comparison of patient demographic, clinical characteristics, treatment, and comorbidities between the groups did not reveal major significant differences except for complement component C3, which was significantly reduced in patients in groups 1 and 3 (figure 5E; appendix p 14). Longitudinal analysis of patient clinical data revealed that patients in group 1 maintained a significantly lower mean concentration of C3, increased mean SLEDAI-2K, and reduced proportion of visits in lupus low disease activity state relative to patients in groups 2–4 (figure 5F–G). This finding was supported by analysis of patient trajectories for SLEDAI-2K and C3, suggesting that patients in group 1 had more active disease over time (figure 5H). Finally, patients in group 1 had a higher mean dose and number of times treated with mycophenolate mofetil relative to patients in groups 2–4 (appendix p 15), as well as increased prevalence (50%) of lupus nephritis. Thus, the juvenile-onset SLE signature distinguished between patients with juvenile-onset SLE and healthy controls, and between patients with longitudinally active and inactive disease.

Finally, the systemic relationships between immunological parameters and serological or clinical biomarkers associated with juvenile-onset SLE were explored using a correlation network analysis. Extensive immune correlations were identified across clinical features (appendix pp 16–17). C3 had a significant negative correlation with total and CD8 EM T cells, supporting the patterns defined with the k-means clustered groups (figure 5; appendix p 14). Disease activity (SLEDAI-2K) correlated negatively with CD4 T cells, naive B cells, and intermediate and non-classical monocytes, and positively with CD8 T cells, and transitional (Bm2') and mature (Bm2) B-cell subsets. Anti-dsDNA antibody measures correlated with early Bm5 and switched memory B cells but not with T-cell subsets. Another notable association was that PDCs negatively correlated with erythrocyte sedimentation rate and C-reactive protein concentrations and positively with haemoglobin concentration.

These results suggest potential interactions between clinical features and disease-related immune dysregulation, which could help to explain the multifactorial, heterogeneous, and systemic nature of the disease.

## Discussion

In this study, machine-learning approaches were applied to analyse immune profiles of patients with juvenile-onset SLE and healthy controls in order to overcome difficulties in obtaining large datasets from cohorts of patients with rare diseases. For the first time, these methodologies have identified and validated an immunological signature associated with juvenile-onset SLE and also distinguished a subgroup of patients with juvenile-onset SLE with more persistently moderate disease activity. Furthermore, network analysis suggested differential relationships between clinical features and specific immune cell subsets in juvenile-onset SLE. Together, these findings improve our understanding of juvenile-onset SLE immunopathology and suggest that immune cell profiles can potentially predict future disease activity.

Although the immunological differences between healthy controls and patients with adult-onset SLE have been established in many previous studies,^17^, ^18^ the immunophenotype of patients with juvenile-onset SLE is less well described. Thus, better understanding of juvenile-onset SLE immune cell defects is imperative considering the increased disease severity seen in patients with juvenile-onset SLE compared with adult-onset SLE overall.

Despite the progress in treatment of juvenile-onset SLE, the long-term outcomes of patients are far from satisfactory. Severity and heterogeneity in presentation, the scarcity of predictive biomarkers for disease activity over time to guide therapeutic decisions, and suboptimal treatment response in selected cases are major obstacles affecting clinical outcomes in patients with juvenile-onset SLE.^2^, ^19^ However, it is increasingly recognised that machine-learning models with substantial predictive accuracy can assist clinicians with complicated therapeutic decision making.^8^, ^9^ Indeed, several studies have used machine-learning methods to analyse complex datasets such as electronic health records,^20^ genetic association and gene expression data,^14^, ^21^, ^22^ and urine biomarkers^15^ to stratify patients with SLE or predict SLE disease activity. Machine-learning applied to immunophenotyping alone has been used to provide insight into disease pathogenesis and to stratify patients with juvenile idiopathic arthritis,^11^ who have also been shown to be distinct from a small number of patients with juvenile-onset SLE used as a comparator. The machine-learning BRF approach used in this study has been used previously to overcome difficulties in obtaining validation datasets in rare cohorts.^23^ This model tends not to suffer from overfitting to training data and by including ten-fold cross-validation, the model performance can be more stringent and less biased compared with models using a simple split of training and test data. Thus, in the absence of separate validation cohorts, using machine learning and cross-validation can help to generate reliable models. This was the case in this study, shown by the high performance of ten-fold cross-validation at 87·8%, suggesting that the identified markers were robust and could enhance current disease classification strategies by providing a more in-depth view of the patient's immunological state. We propose that such an analysis pipeline could be applied to other independent cohorts to help define unique, cohort-specific patient subgroups associated with differential clinical outcomes.

The machine-learning analysis pinpointed eight of the 28 immune cell subtypes examined to be consistently associated with patients with juvenile-onset SLE compared with healthy controls. Furthermore, the B-cell phenotype was stable across all patients with juvenile-onset SLE, which might explain the increased predictive power of these subsets in the BRF model, whereas the CD8 T-cell phenotype was important in stratifying patients with juvenile-onset SLE. The B-cell signature included reduced frequencies of naive (Bm1) and unswitched memory B cells, suggesting that patients with juvenile-onset SLE had a more mature memory B-cell phenotype. Few studies have specifically examined the B-cell phenotype in patients with juvenile-onset SLE, and although B cells in general are dysregulated in patients with SLE,^24^ these defects are more pronounced in patients with adult-onset SLE.^25^ Notably, CD27++ plasmablast frequencies are elevated in patients with juvenile-onset SLE,^25^ and patients have distinct plasmablast transcriptomic profiles,^10^ supporting increased B-cell activation in juvenile-onset SLE. T-cell abnormalities were also dominant in our identified juvenile-onset SLE signature, in particular increased total and naive CD8 T cells and reduced total CD4 and CD8 EM frequencies, as well as reduced invariant natural killer T-cell frequencies, as previously reported in adult-onset SLE and juvenile-onset SLE.^11^ Pro-inflammatory T-cell profiles have been described in patients with juvenile-onset SLE and active lupus nephritis.^26^, ^27^ However, no changes in regulatory T cells were identified in this study in contrast to the reported increased CD4 effector and decreased regulatory T-cell frequencies in patients with juvenile-onset SLE and lupus nephritis.^26^

A striking feature of this study was the differential CD8 T-cell profile, which contributed to both the juvenile-onset SLE signature and stratification of juvenile-onset SLE patient subgroups. In particular, patients with juvenile-onset SLE who had elevated CD8 EM T-cell frequencies were associated with increased disease activity at baseline and tended towards a more active disease trajectory (and in particular, a higher proportion of lupus nephritis), supporting a role for CD8 T cells in the pathogenesis of juvenile-onset SLE. Expanded CD8 memory T cells have been associated with poor disease outcome measures when combined with transcriptional profiling in adults with SLE.^28^, ^29^ Previous research suggested that CD8 EM T cells have decreased proliferative capacity and a strong inclination towards apoptosis in SLE, while expressing high levels of interferon-γ, granzyme B, and perforin, thus potentially contributing to chronic inflammation and organ damage.^30^ However, the mechanisms driving increased memory CD8 T cells in juvenile-onset SLE remain uncertain and require further investigation.

This study has some limitations. As disease in the majority of our patients with juvenile-onset SLE was reasonably well controlled by medication, investigation of severely active juvenile-onset SLE phenotype was not possible. Furthermore, SLEDAI-2K, as all disease activity scores, is an imperfect measure of changes in disease severity; however, despite its limitations, the SLEDAI-2K score is widely used in clinical practice to guide therapeutic decisions and define SLE activity categories and was applied consistently when assessing the patients longitudinally. Notably, mycophenolate mofetil doses were significantly elevated in group 1, and this elevation was probably a reflection of the higher disease activity in group 1 patients than in patients in groups 2–4. Furthermore, although longitudinal clinical data were collected, we cannot account for patient non-compliance to prescribed therapy (eg, mycophenolate mofetil levels were not tested), which could affect the analysis comparing phenotype with disease outcomes and treatment. In addition, no organ-specific immune analysis was done because of practical reasons. Children and adolescents with juvenile-onset SLE are diagnosed on the basis of expert opinion and classified using adult-tailored classification criteria (the American College of Radiology and SLICC classification criteria). This approach can pose considerable challenges in diagnosing patients with atypical presentations. As a consequence, many patients with juvenile-onset SLE are initially labelled as having arthritis or myositis, which have been identified as manifestations of juvenile-onset SLE. For this reason, we have not excluded patients with a concomitant diagnosis of arthritis or myositis from this study. Another characteristic of this juvenile-onset SLE cohort was the small number of patients with black ethnicity within this cohort and specifically within group 1; ethnicity could play a part in altered immune cell phenotype between the groups, as has been shown previously.^31^ The imbalance in the sex and ethnicity between healthy controls and patients with juvenile-onset SLE is a practical problem limited by the willingness of adolescent healthy donors to participate in research. Furthermore, machine-learning algorithms function like a black box that produces reasonable prediction results without giving complementary justification. Thus, it would be important for this analysis to be validated in external cohorts and done on a cohort by cohort basis to account for ethnicity changes between regions or countries and to prospectively validate the association between immune cell subsets and disease progression.

In conclusion, the application of machine-learning approaches to immune phenotyping data has identified immunological biomarkers that could potentially help to unravel underlying disease mechanisms in juvenile-onset SLE and explain the differences in long-term outcomes of patients with this disease. Such immunological signatures could facilitate better stratification of patients for optimal treatment choices and provide information to improve interventional clinical trial design.
