# Supplementary material for: Disease-associated and patient-specific immune cell signatures in juvenile-onset systemic lupus erythematosus: patient stratification using a machine-learning approach
Source: Lancet Rheumatol. 2020 Jul 29;2(8):e485–96. doi: 10.1016/S2665-9913(20)30168-5 (PMC7425802; doi:10.1016/S2665-9913(20)30168-5)
Supplement: Supplementary appendix [file mmc1.pdf]

THE LANCET
Rheumatology

Supplementary appendix

This appendix formed part of the original submission and has been peer reviewed. We post it as supplied by the authors.

Supplement to: Robinson GA, Peng J, Dönnes P, et al. Disease-associated and patient-specific immune cell signatures in juvenile-onset systemic lupus erythematosus: patient stratification using a machine-learning approach. *Lancet Rheumatol* 2020; **2:** e485–96.

PATIENTS AND CONTROLS

Study protocol excerpt: Extracted from Adolescent Centre for Rheumatology – Centre Ethics protocol document (REC no. Ref.11/LO/0330).

Patient cohort:

Eligibility and recruitment:

Inclusion:

- An autoimmune rheumatic disease fulfilling internationally recognised consensus classification criteria. Patients with juvenile systemic erythematosus (JSLE) should fulfil the 1997 American College of Rheumatology (ACR)[1] or/and the 2012 Systemic Lupus International Collaborating Clinics (SLICC)[2] classification criteria, and be diagnosed before 18 years of age.
- For controls, volunteers who are either healthy or with non-inflammatory, non-infective conditions (e.g. referred for assessment of non-inflammatory musculoskeletal conditions)
- Aged 6 years or older
- Puberty tanner stage 4-5

Exclusion:

- Any patient who withholds consent or whose carer withholds consent (as appropriate given patient's competence)
- Any patient who withdraws from the study

Additional criteria for this paper

Inclusion: JSLE samples with more than 10 million PBMCs/sample. Patients and controls can only be included on one occasion.
Exclusion: patients treated with Rituximab or Cyclophosphamide in the last 12 months

Sample size: This was an exploratory study based on the number of patients and healthy donors available fitting the inclusion/exclusion criteria. Patients were included at one time point only– no longitudinal blood samples collected. The data provided here will provide a sound basis for future work.

Justification for not excluding JSLE patients with overlapping clinical phenotypes
Children and adolescents with JSLE are diagnosed based on expert opinion and classified using adult-tailored classification criteria (the ACR and SLICC classification criteria). This can pose significant challenges in diagnosing patients who do not fulfil adult classification criteria at presentation. As a consequence, many JSLE patients have initially been labelled as having arthritis or myositis, which ulterior have been identified as manifestations of JSLE. For this reason, we have not excluded patients with a concomitant diagnosis of arthritis or myositis from this study. We included patients with anti-phospholipid antibodies associated with JSLE but no features of anti-phospholipid syndrome.

Demographic, Clinical and Treatment data collected at baseline and after longitudinal follow-up. Demographics (age, sex, ethnicity, BMI, disease duration); Serology (dsDNA, antinuclear antibodies, extractable nuclear antigens, CRP, C3, lymphocyte count, neutrophil count, urine protein: creatinine, haemoglobin, platelet count); Organ involvement (renal, central nervous system, cardiovascular, musculoskeletal, haematological, gastrointestinal, skin); Co-morbidities; Disease activity scores SLE Disease Activity Index-2000 (SLEDAI-2000)[3], SLICC[2], Lupus Low Disease Activity State (LLDAS)[4]; Treatment (Hydroxychloroquine, Mycophenolate mofetil, Prednisolone, Vitamin D, Methotrexate, Azathioprine, Rituximab, Cyclophosphamide, intravenous immunoglobulin).

FLOW CYTOMETRY

Markers used to identify cell types for immunophenotyping by flow cytometry

	Cell type	Markers
**T-cells**	CD4 T-cell	CD3+, CD4+
	CD8 T-cell	CD3+, CD8+
	Naïve CD4 T-cell	CD4+, CD27+, CD45RA+
	Central memory (CM) CD4 T-cell	CD4+, CD27+, CD45RA-
	Effector memory (EM) CD4 T-cell	CD4+, CD27-, CD45RA-
	Effector memory (EM) RA CD4 T-cell	CD4+, CD27-, CD45RA+
	Naïve CD8 T-cell	CD8+, CD27+, CD45RA+
	Central memory (CM) CD8 T-cell	CD8+, CD27+, CD45RA-
	Effector memory (EM) CD8 T-cell	CD8+, CD27-, CD45RA-
	Effector memory (EM) RA CD8 T-cell	CD8+, CD27-, CD45RA+
	Regulatory T-cell (Treg)	CD4+, CD25+, CD127-
	Tresponder T-cell (Tresp)	CD4+, CD25-, CD127+
	Invariant natural killer T- (iNKT) cells	CD3+, iTCR+
**B-cells**	B-cells	CD19+
	Bm1 (naïve)	IgD+, CD38-
	Bm2 (mature)	IgD+, CD38+
	Bm2' (Transitional)	IgD+, CD38++
	Bm3-4 (plasmablasts)	IgD-, CD38++
	Early Bm5 (early memory)	IgD-, CD38+
	Late Bm5 (late memory)	IgD-, CD38-
	Naïve	IgD+, CD27-
	Unswitched memory	IgD+, CD27+
	Switched memory	IgD-, CD27+
**Monocytes**	Monocytes	CD14+
	Classical	CD14+, CD16-
	Non-classical	CD14+, CD16+
	Intermediate	CD14-, CD16+
**PDC**	Plasmacytoid dendritic cell (PDC)	CD303+

Markers used to identify cell types for immunophenotyping by flow cytometry. List of markers used to define all 28 immune cell subsets (T-cells, B-cells, monocytes and PDCs) used in the paper analysis. These markers were targeted by antibodies described in Appendix p 3.

Table of antibodies used for flow cytometry

Fluorochrome	T-cells	Company	Clone	APCs	Company	Clone
**UV 350⁄450**	Fixable Blue Dead Stain	ThermoFisher Scientific	N/A	Fixable Blue Dead Stain	ThermoFisher Scientific	N/A
**BUV395**	CD4	BD	SK3	CD19	BD	SJ25C1
**AF700**	CD27	Biolegend	M-T271			
**BV421**	CD8a	Biolegend	RPA-T8	CD38	Biolegend	HB-7
**BV510**				IgD	Biolegend	IA6-2
**BV711**	CD127	Biolegend	A019D5	CD14	Biolegend	M5E2
**BV785**	CD3	Biolegend	OKT3	HLA-DR	Biolegend	L243
**PERCP Cy5.5**				CD303	Biolegend	201A
**PE**	TCR Vα24-Jα18 (iNKT)	Biolegend	6B11			
**PE-Dazzle594**	CD25	Biolegend	M-A251	CD16	Biolegend	3G8
**PE-CY7**	CD45RA	Biolegend	HI100	CD27	Biolegend	M-T271

Table of antibodies used for flow cytometry
Antibodies used for immunophenotyping by flow cytometry. Two panels were developed, one for T-cells and one for antigen presenting cells (APCs). Target marker, conjugated fluorochrome, company and clone are displayed.

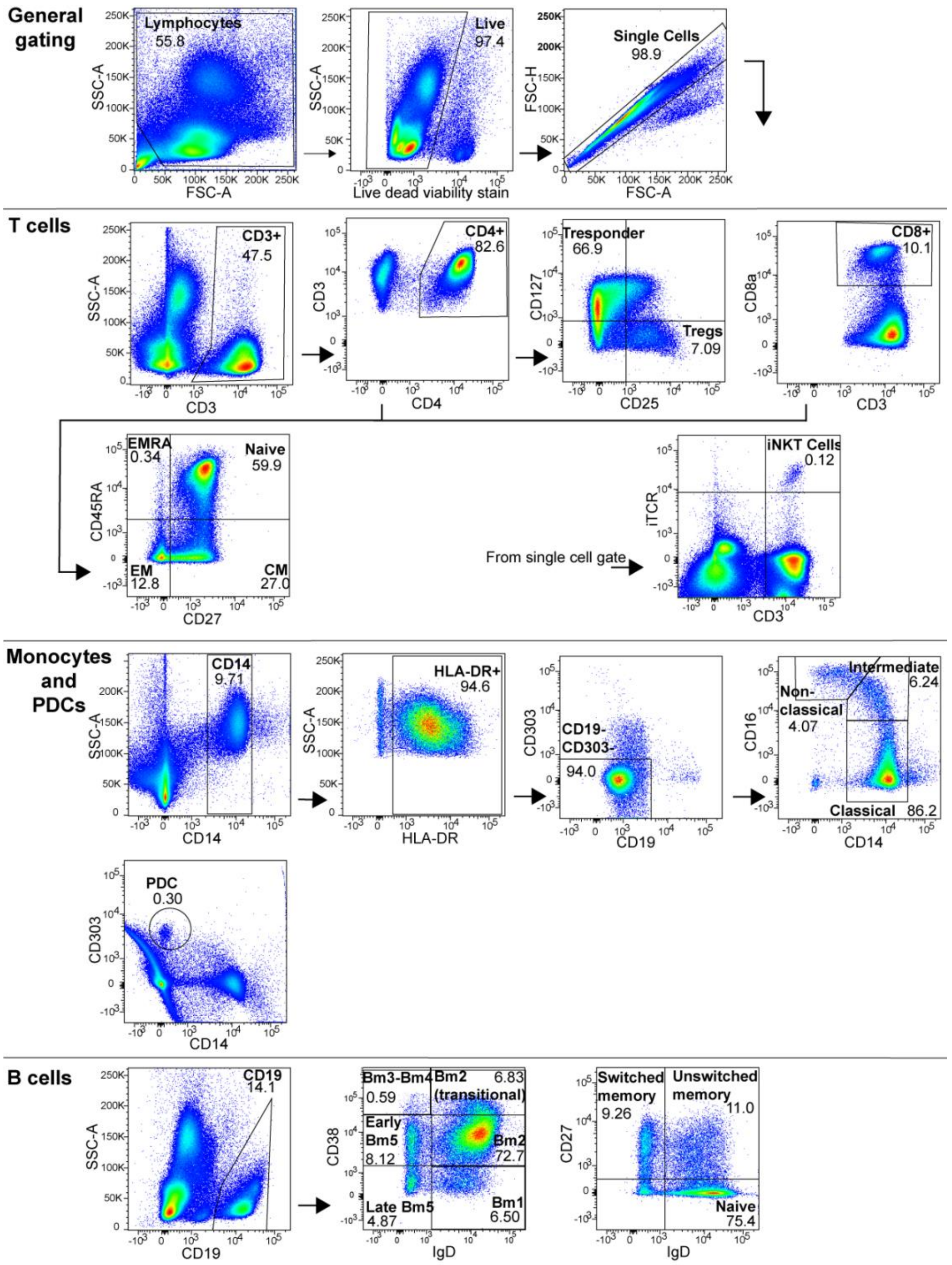

Gating strategy for immune cell identification: Representative gating strategies from a healthy donor used to identify T-cell, B-cell, Monocyte and PDC subsets. PBMC’s were stained with fluorescently labelled antibodies and measured by flow cytometry. Labels represent the cell population within the gate and the percentage of it’s parent gate. Abbreviations: Regulatory T-cells (Tregs), invariant natural killer T-cells (iNKT-cells), central memory (CM), effector memory (EM), plasmacytoid dendritic cell (PDC), Bm1 (naïve), Bm2 (mature), Bm2’ (transitional), Bm3-4 (Plasmablasts), early/late Bm5 (memory). Refer to Figure 2.

DATA ANALYSIS

T-Tests: Data was corrected for multiple testing by two-stage linear step-up procedure of Benjamini, Krieger and Yekutieli (false discovery rate- FDR- of 5%)[5].

Detailed description of ML and LR approaches used

Correlation Comparison analysis: Spearman correlation tests between pairs of immune cell types (n=28) in both HC and JSLE patients were performed using R version 3.5.2[6]. The significance for the difference in corresponding correlation between HC and JSLE patients was calculated using the cocor package in R (*cocor.indep.groups* function)[7]. Spearman correlation coefficients for pairs of immune cell types in HC and JSLE were Symmetrically plotted in a heat map using the heatmap.2 function from gplots package in R[8]. The correlation coefficients with significant difference ($p<0.05$ and 0.01) were highlighted in Figure 1.D and Appendix p.9.

Balanced random forest (BRF): To stratify JSLE patients from the HC using immunophenotyping data, the balanced random forest (BRF) approach was used with the randomForest package in R[9]. A balanced random forest (BRF) is an ensemble ML algorithm for classification, consisting of numerous decisions trees which can increase model accuracy without the risk of model overfitting[10] which is often a problem when analysing data with small sample size. In addition, the predictive performance of the BRF model can be estimated and assessed by 10-fold cross validation which mitigates the need for independent validation, giving an advantage when investigating rare patient cohorts.

Decision trees were built using a bootstrap dataset consisting of randomly selected samples from the original dataset (n=106), allowing the same sample to be selected more than once. As the original sample set had an unbalanced HC:JSLE (39:67) ratio, the balanced method was applied in the bootstrap dataset construction. The bootstrap dataset was first selected from the minority class (HC, n=39) whilst randomly drawing the exact number (n=39) from the majority class (JSLE). The balanced bootstrap dataset (n=39 HC and n=39 JSLE; total n=78) was then used for model training. After creating the bootstrap dataset, only a random subset of immunological variables was considered at each split of the decision tree. Every decision tree was built by constructing a new bootstrap dataset and considering a newly selected subset of variables at each step. A total 10,000 decision trees were used for the BRF model construction, allowing the output to be stabilised and to ensure the reliable predictive performance of the model. The classification output of the model was provided by aggregating the predictions of every decision tree and making the final prediction. Samples that were not included in the bootstrap dataset were termed the Out-of-Bag (OOB)

dataset and were used to validate the model performance. Demographic factors were included into the BRF model for adjustment purposes.

Model optimisation testing was performed to determine the exact number of immunological variables ($N_{variable}$) included in each subset for building the decision tree. By comparing the accuracy of the BRF model with different Nvariable settings ($N_{variable}$ range 1-28), the model with the overall lowest classification error rate was selected as the optimal model and applied in further analysis; in this case 10 immunological parameters per split ($N^{variables}$=10) gave the best overall model accuracy. For model performance evaluation, the receiver operator characteristic (ROC) plot and the area under the curve (AUC) of each model was computed with the pROC package in R[11].

Sparse partial least squares discriminant analysis (sPLS-DA): This supervised ML approach was operated using the mixOmics packagein R[12]. Ten-fold cross-validation with 50 repetitions was applied to prevent model overfitting. Model optimisation was applied to select the number of components included in the sPLS-DA model. Models with different component numbers were assessed by 10-fold cross-validation x10, using the overall error rate (blue line) and balanced error rate (BER, yellow line) to evaluate model performance. The models with four components gave the lowest overall estimation error rate (16.7%) and BER (17.5%) and were selected as optimal, giving the best discriminatory performance for further analysis. The separation of JSLE and HC samples was presented by projecting the samples into the subspace constructed of component 1 and component 2. The prediction interval of the model was calculated from the 95% confidence ellipses for the HC group and the JSLE group. The top 10 weighted immunological parameters were selected and presented by variable loading plots.

Model validation: 10-fold cross validation for BRF and sPLS-DA was applied with the caret package[13] and mixOmics package[12] in R, respectively. Data were randomly partitioned into 10 groups with almost equal size. Nine groups were used as training data for model construction and the remaining group was used as validation data. The process was repeated for all 10 folds until each observation in the data is used for validation purpose once. The average performance of the 10 models was used as the result of the 10-fold cross validation.

Logistic regression for association analysis: The association between the immunophenotypes of 28 parameters and JSLE was assessed by univariate logistic regression analysis adjusted for age, sex and ethnicity. For each measurement, the odds ratio (OR) and the 95% confidence interval (CI) were determined. The p-value for each association was calculated in the logistic regression analysis. Forest plots produced with

the ggplot2 package in R[14] were used to present the logistic regression analysis results, with significant associations highlighted in blue ($p < 0.05$).

K-means clustering analysis: Performed with the stats package in R[15]. The k-means clustering algorithm repositions the specific amount of cluster centroids around the JSLE samples (n=67) until the most convergent grouping appears. The number of groupings in k-means clustering is determined by the elbow method (see figure below). Immune-phenotypes were standardised and displayed as a heatmap.

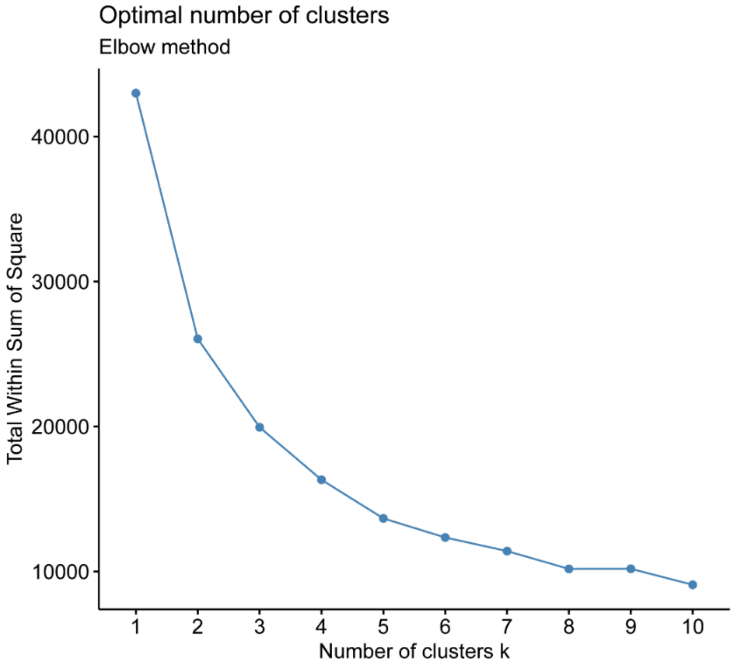

The Elbow method was applied to help decide on the appropriate number of clusters for K-means clustering. Y-axis: The total within-cluster sum of square (WSS), measures the compactness of the clustering; X-axis: Number of clusters (n=1 to 10). The WSS decreases gradually so that no distinct turning point can be identified.

Clinical trajectory analysis: Trajectory of patient clinical measures over time (visits, n=25) were depicted by Spaghetti plot. The flow of the longitudinal data of JSLE patients (n=67) were shown in each plot where each line represents one parameter from each JSLE patient. Smoothing lines were added to indicate the trend of JSLE groups from previous k-means clustering. Plots produced using R package “ggplot2”.

Network analysis: Performed using Force Atlas layout in Gephi[16]. 16 clinical features, namely SLEDAI score, cholesterol, high-density lipoprotein (HDL), C:HDL, low-density lipoprotein (LDL), triglycerides, lymphocyte count, complement component 3 (C3), C-

reactive protein (CRP), double-stranded DNA (dsDNA), erythrocyte sedimentation rate (ESR), haemoglobin, platelet count, urine protein:creatinine ratio, neutrophil count and body mass index (BMI) were applied in the network analysis. Pearson correlation coefficients for each association were calculated in R. Only correlations with an absolute value of ≥0.2 are shown in the graph.

Software and packages used in analysis

Software/Package	Version	Purpose in this project	References
'caret' package	6.0-84	**10-fold cross validation**	Kuhn, M. (2012). *The caret package.* R Foundation for Statistical Computing, Vienna, Austria. URL https://cran. r-project. org/package= caret.
'cocor' package	1.1-3	**Correlation comparison**	Diedenhofen, B. and Musch, J. (2015). cocor: *A Comprehensive Solution for the Statistical Comparison of Correlations*. PLOS ONE, 10(4), p.e0121945.
Gephi	0.9.2	**Network analysis**	Bastian, M., Heymann, S. and Jacomy, M., (2009). March. *Gephi: an open source software for exploring and manipulating networks.* In Third international AAAI conference on weblogs and social media.
'ggplot2' package	3.2.1	**Produce plots**	Wilkinson, L. (2011). *ggplot2: Elegant Graphics for Data Analysis by WICKHAM, H.* Biometrics, 67(2), pp.678-679.
'gplots' package heatmap.2	3.0.1.1	**Produce heatmaps**	Warnes, M.G.R., Bolker, B., Bonebakker, L. and Gentleman, R., (2016). *Package 'gplots'*. Various R Programming Tools for Plotting Data.
'mixOmics' package	6.6.2	**sPLS-DA analysis**	Rohart, F., Gautier, B., Singh, A. and Lê Cao, K.A., (2017). mixOmics: *An R package for 'omics feature selection and multiple data integration.* PLoS computational biology, 13(11), p.e1005752.
'pROC' package	1.15.0	**Produce ROC plot and calculate AUC**	Robin, X., Turck, N., Hainard, A., Tiberti, N., Lisacek, F., Sanchez, J.C. and Müller, M., (2011). *pROC: an open-source package for R and S+ to analyze and compare ROC curves*. BMC bioinformatics, 12(1), p.77.
'randomForest' package	4.6-14	**balanced random forest model**	Liaw, A. and Wiener, M., (2002). *Classification and regression by randomForest*. R news, 2(3), pp.18-22.
R Studio	1.1.463	**Statistical analysis**	Racine, J.S., (2012). *RStudio: a platform-independent IDE for R and Sweave. Journal of Applied Econometrics*, 27(1), pp.167-172.
'stats' package	3.5.2	**k-means clustering analysis**	R Core Team. (2018). *R: A language and environment for statistical computing*.

Software and packages used in analysis
Software packages used in R software throughout the project analysis. The name of the package, version, purpose and reference are displayed.

JSLE\HC	Tresp	Bm1	CD19 + Unswi tched memo ry	iNKT	CD8+	Classi cal	CD19 +	Bm2 (Tran sition al)	Bm2	CD19 + Naive	CD4+ Naive	CD8+ Naive	CD4+	CD14 +	PDC's	Late Bm5	Early Bm5	CD19 + Switc hed memo ry	CD8+ EM	CD8+ EMR A	CD4+ EM	CD4+ EMR A	Inter media te	non- classi cal	CD4+ CM	CD8+ CM	Treg	Bm3- Bm4
Tresp	1	0.35	0.36	0.06	-0.09	0.15	0.09	-0.11	-0.19	-0.27	0.02	0.07	0.06	0.05	0.32	0.14	0.04	0.12	-0.05	-0.16	0.02	-0.24	0.23	-0.18	0.11	0.2	-0.42	-0.08
Bm1	0.05	1	0.71	0.08	0.15	0.34	-0.33	-0.54	-0.66	-0.6	0.03	0.14	-0.15	0	0.21	0.44	0.14	0.32	0.03	-0.28	-0.2	-0.1	-0.28	-0.35	0.14	0.32	-0.2	0.03
CD19+ Unswitched memory	-0.03	0.59	1	-0.01	0.04	0.44	-0.26	-0.4	-0.42	-0.7	0.07	0.11	0.04	0.06	0.19	0.13	0.18	0.3	-0.08	-0.25	-0.27	-0.26	-0.23	-0.36	0.13	0.32	-0.03	0.16
iNKT	0.2	-0.17	-0.29	1	0.04	0.15	-0.08	-0.19	-0.05	-0.02	-0.08	0.22	-0.03	-0.06	0.2	-0.03	0.07	-0.01	-0.05	-0.14	-0.09	-0.06	-0.22	-0.08	0.15	-0.05	-0.28	-0.04
CD8+	-0.16	-0.25	-0.19	0.07	1	0.24	-0.19	0.15	0.02	0.09	-0.14	0.02	-0.91	-0.03	-0.11	-0.19	-0.15	-0.2	-0.12	0.13	0	0.23	-0.31	-0.28	0.16	-0.29	-0.01	0.06
Classical	-0.1	0.03	-0.01	-0.11	-0.15	1	0	0.06	-0.04	-0.09	0.17	0.51	-0.08	0.31	0.23	-0.24	-0.17	-0.18	-0.32	-0.36	-0.24	-0.14	-0.53	-0.95	0.03	-0.05	-0.26	0.03
CD19+	0.02	-0.06	-0.29	0.01	-0.23	0.06	1	0.33	0.4	0.4	0.27	0.3	0.24	0.03	-0.12	-0.3	-0.31	-0.32	-0.39	-0.18	-0.31	-0.17	0.06	-0.07	-0.2	-0.19	-0.07	-0.24
Bm2 (Transitional)	-0.14	-0.57	-0.44	-0.03	0.21	-0.15	0.28	1	0.57	0.65	0.14	0.24	-0.03	0.18	-0.08	-0.65	-0.53	-0.61	-0.29	-0.11	-0.02	-0.01	0.21	-0.14	-0.11	-0.26	0.25	0.03
Bm2	0.05	-0.43	-0.51	0.26	-0.1	-0.06	0.4	0.3	1	0.84	0.17	0.08	0	0.18	0.04	-0.82	-0.73	-0.83	-0.12	0.05	-0.11	-0.07	0	0.01	-0.19	-0.25	0.04	-0.42
CD19+ Naive	-0.06	-0.51	-0.69	0.29	0.03	-0.05	0.47	0.61	0.84	1	0.08	0.14	-0.11	0.21	0.03	-0.69	-0.75	-0.85	-0.1	0	-0.03	0.04	0.06	0.02	-0.13	-0.28	0.04	-0.37
CD4+ Naive	0.19	0.12	0.16	-0.06	-0.19	-0.1	-0.18	-0.23	-0.01	-0.16	1	0.62	0.25	0.09	0.17	-0.12	-0.3	-0.11	-0.43	-0.37	-0.65	-0.45	-0.03	-0.21	-0.83	-0.17	-0.19	-0.14
CD8+ Naive	0.09	0.16	0	-0.08	-0.25	0.05	0.01	-0.25	0.11	-0.06	0.4	1	0.08	0.25	0.14	-0.33	-0.31	-0.26	-0.66	-0.75	-0.64	-0.41	-0.18	-0.5	-0.28	-0.14	-0.19	-0.04
CD4+	0.31	0.2	0.2	-0.13	-0.92	0.11	0.22	-0.24	0.11	-0.08	0.32	0.31	1	0.11	0.11	0.13	0.1	0.16	0	-0.16	-0.07	-0.2	0.14	0.11	-0.26	0.2	0.01	-0.08
CD14+	-0.09	0	-0.3	0.02	-0.21	0.33	0.07	-0.03	0.27	0.28	-0.14	0.05	0.13	1	0.42	-0.27	-0.21	-0.24	0.01	-0.36	0.01	0.04	-0.11	-0.28	-0.1	0.17	-0.11	-0.15
PDC's	-0.06	0.06	0.15	-0.1	-0.3	-0.18	0.1	-0.09	0.25	0.02	-0.17	-0.03	0.31	0.15	1	-0.14	-0.1	-0.13	0.03	-0.36	-0.2	-0.28	-0.03	-0.17	-0.03	0.25	-0.35	-0.01
Late Bm5	0.16	0.64	0.51	-0.34	-0.15	0.07	-0.31	-0.61	-0.72	-0.85	0.16	0.13	0.2	-0.13	-0.02	1	0.67	0.83	0.31	0.26	0.3	0.25	0.05	0.25	0	0.15	-0.08	0.21
Early Bm5	0.1	0.35	0.55	-0.27	0.08	0.01	-0.45	-0.52	-0.82	-0.95	0.15	-0.01	-0.01	-0.28	-0.09	0.76	1	0.89	0.24	0.15	0.29	0.17	0.15	0.26	0.21	0.14	0	0.38
CD19+ Switched memory	0.11	0.45	0.7	-0.31	-0.05	0.06	-0.44	-0.57	-0.74	-0.93	0.21	0.01	0.12	-0.28	0.02	0.82	0.89	1	0.19	0.11	0.21	0.07	0.13	0.25	0.07	0.21	0.03	0.43
CD8+ EM	-0.04	-0.2	-0.04	0.07	0.28	0	0.05	0.22	-0.07	0.05	-0.45	-0.83	-0.3	-0.08	0.03	-0.12	0	0.02	1	0.4	0.61	0.45	0.18	0.28	0.12	0.18	0.24	0.05
CD8+ EMRA	-0.04	-0.12	0.11	0.1	0.21	-0.06	-0.19	0.09	-0.1	-0.08	-0.13	-0.78	-0.19	-0.15	0.08	-0.03	0.14	0.14	0.58	1	0.58	0.48	-0.07	0.37	-0.06	-0.36	0.17	-0.07
CD4+ EM	-0.19	-0.23	0	0.14	0.2	0.03	-0.06	0.1	-0.01	-0.01	-0.58	-0.58	-0.25	0.1	0.29	-0.11	0.05	0	0.65	0.48	1	0.7	0.12	0.24	0.24	-0.18	0.23	0.08
CD4+ EMRA	-0.15	-0.21	0.07	0.06	0.13	0.06	-0.1	0.06	-0.05	-0.06	-0.15	-0.49	-0.15	0.01	0.16	-0.05	0.13	0.08	0.43	0.58	0.65	1	-0.1	0.14	0.07	-0.35	0.24	0.07
Intermediate	0.01	-0.17	-0.18	0.21	0.19	-0.48	-0.15	0.34	0.22	0.31	-0.07	-0.15	-0.16	0.17	0.04	-0.35	-0.27	-0.32	-0.03	0.09	0	-0.06	1	0.47	0.01	0.26	0.15	0.31
non-classical	0.17	0.04	-0.02	0.08	-0.09	-0.75	0.09	0.15	0.22	0.17	0.08	-0.01	0.12	-0.13	0.32	-0.09	-0.13	-0.15	-0.1	0.01	-0.12	-0.14	0.48	1	0.03	0.08	0.3	0.04
CD4+ CM	-0.13	-0.1	-0.2	0.02	0.13	0.12	0.26	0.25	0.04	0.21	-0.91	-0.22	-0.27	0.14	0.06	-0.16	-0.2	-0.24	0.28	-0.03	0.31	-0.09	-0.01	-0.09	1	0.43	0.14	0.21
CD8+ CM	0.06	0.04	-0.09	0.12	0.03	-0.03	0.26	0.2	0.01	0.18	-0.45	-0.53	-0.2	0.11	0.03	-0.12	-0.18	-0.15	0.57	0.02	0.3	-0.01	0.02	0.02	0.47	1	0.11	0.15
Treg	-0.41	-0.29	-0.07	-0.25	0.18	0.1	-0.1	0.24	0.03	0.16	-0.27	-0.18	-0.25	0.1	0.06	-0.24	-0.1	-0.2	0.11	0.14	0.32	0.21	0.17	0.02	0.21	-0.05	1	0.42
Bm3-Bm4	-0.03	0.03	0.19	-0.1	0.31	-0.11	-0.48	-0.05	-0.39	-0.38	-0.05	0.01	-0.33	-0.16	-0.07	0.24	0.41	0.39	0	-0.07	0.12	-0.06	0.19	-0.04	-0.03	0.01	0.09	1

Correlation comparison analysis between HCs and JSLE patients

Correlation comparison analysis was performed using R software ('cocor' package) on 28 immune cell subsets from 39 HCs and 67 JSLE. **Top right** of table: HC spearman coefficients and values are displayed in red when the correlation was significant following bonferroni correction. **Bottom left** of table: JSLE spearman coefficients displayed and values are boxed in red where a significant difference was observed when compared to HCs ($p<0.05$, white text $p<0.01$). Refer to Figure 2D.

Odds ratio from logistic regression analysis

Immune cell	Odds ratio	Mean	95% CI (lower)	95% CI (upper)	p value
CD19+ Unswitched memory	7.07E-01	0.7069949	0.60380823	0.8278155	0.00002
Bm1	7.95E-01	0.7950473	0.70377505	0.8981566	0.00023
CD14+	1.32E+00	1.3176686	1.12169761	1.5478775	0.00079
CD8+ CM	8.93E-01	0.8925118	0.82878695	0.9611364	0.00262
CD8+ Naive	1.07E+00	1.0680346	1.02247846	1.1156205	0.00308
CD4+ EM	8.40E-01	0.8398257	0.74319662	0.9490186	0.00513
CD8+	1.09E+00	1.0889428	1.0228551	1.1593004	0.00764
CD8+ EM	7.69E-01	0.7692921	0.63268518	0.9353948	0.00855
iNKT	4.59E-06	4.59E-06	1.52E-10	0.1380834	0.01948
CD4+	9.42E-01	0.9417829	0.89481056	0.9912209	0.02157
PDC's	2.03E-02	0.0202740	0.00061073	0.6730257	0.02914
CD19+ Naive	1.04E+00	1.0432759	1.0038377	1.0842636	0.03118
Bm2 (Transitional)	1.19E+00	1.1937565	0.99563634	1.4313002	0.05578
CD4+ Naive	1.05E+00	1.0496237	0.99551113	1.1066776	0.07291
Treg	1.33E+00	1.3343135	0.97158121	1.8324692	0.07477
CD4+ EMRA	6.77E-01	0.6767741	0.43699419	1.0481219	0.08022
Bm3-Bm4	1.98E+00	1.9795842	0.87575411	4.4747190	0.10076
CD19+	1.12E+00	1.1243854	0.97661147	1.2945193	0.10293
non-classical	9.17E-01	0.9168064	0.81993096	1.0251278	0.1274
Classical	1.06E+00	1.0554703	0.97428974	1.1434150	0.18613
Tresp	9.62E-01	0.9617618	0.90386575	1.0233663	0.21839
Late Bm5	9.33E-01	0.9332045	0.83481501	1.0431900	0.22393
CD19+ Switched memory	9.65E-01	0.9653879	0.90629071	1.0283386	0.27441
CD8+ EMRA	9.77E-01	0.9768912	0.93281488	1.0230501	0.32093
Intermediate	1.10E+00	1.0988609	0.83937212	1.4385697	0.49275
Bm2	1.01E+00	1.0141421	0.97118762	1.05899647	0.52479
Early Bm5	1.02E+00	1.0195914	0.950295	1.09394096	0.589
CD4+ CM	9.90E-01	0.989682	0.92725386	1.05631306	0.75504

Odds ratio from logistic regression analysis
Logistic regression analysis was performed comparing 28 immune cell subsets between 39 HCs and 67 JSLE patients. Odds ratios, means, 95% confidence intervals and p values are displayed are displayed and significant immunological parameters are shown in red. Gender, ethnicity and age were adjusted in the logistic regression analysis to avoid confounding effects. Refer to Figure 4A.

Selection of important immunological features from ML analysis

	Multiple t-test (corrected)	BRF	sPLS-DA	Logistic regression
Total CD4+	✓	✓	✓	✓
CD4+ CM				
CD4+ EM	✓	✓		✓
CD4+ EMRA	✓			
CD4+ Naive			✓	
Total CD8+	✓	✓	✓	✓
CD8+ CM	✓		✓	✓
CD8+ EM	✓	✓	✓	✓
CD8+ EMRA	✓			
CD8+ Naive	✓	✓	✓	✓
iNKT	✓	✓	✓	✓
Treg				
Tresp				
CD19+				
Bm1	✓	✓	✓	✓
Bm2				
Bm2 (Transitional)	✓			
Bm3-Bm4	✓			
Early Bm5				
Late Bm5				
CD19+ Naive		✓		✓
CD19+ Switched memory				
CD19+ Unswitched memory	✓	✓	✓	✓
CD14+	✓	✓	✓	✓
Classical				
Intermediate				
non-classical				
PDC's	✓		✓	✓

Selection of important immunological features from ML analysis: 28 immune cell subsets detected by immunopenotyping PBMCs from HCs and JSLE patients assessed by flow cytometry and analysed using multiple ML models. These are listed and ticked where: **Column 1)** selected as significant by multiple t-tets corrected for multiple testing, 5% FDR (Benjamini, Krieger and Yekutieli) **Column 2)** selected as top 10 most important variables in the balanced random forest (BRF) model; **Column 3)** selected as top 10 weighted variables in sparse partial least squares discriminant analysis (sPLS-DA); **Column 4)** significantly associated with JSLE in logistic regression analysis. Markers highlighted in green are those recognised by all strategies. Abbreviations: Regulatory T-cells (Tregs), invariant natural killer T-cells (iNKT-cells), central memory (CM), effector memory (EM), plasmacytoid dendritic cell (PDC), Bm1 (naïve), Bm2 (mature), Bm2’ (transitional), Bm3-4 (Plasmablasts), early/late Bm5 (memory).

Summary of the JSLE immune cell signature

		HC		JSLE		p value
		Mean	SD	Mean	SD	
T cells	CD4+	74.68205	7.563307	66.38806	13.27934	0.0016
	CD4+ CM	33.62308	8.246354	35.35522	10.39473	0.2401
	CD4+ EM	8.325897	4.299036	6.134179	4.526059	0.0181
	CD4+ EMRA	1.114513	2.146454	0.421493	0.784872	0.0196
	CD4+ Naïve	56.94872	10.36073	58.08955	12.04973	0.3659
	CD8+	17.11436	7.230942	25.54851	12.22465	0.0006
	CD8+ CM	19.65256	8.592842	14.09507	7.859905	0.0024
	CD8+ EM	5.484615	3.120663	3.798209	3.32964	0.0163
	CD8+ EMRA	16.15128	16.75405	10.58896	10.66448	0.0383
	CD8+ Naïve	58.72564	16.24401	71.51493	14.7692	0.0005
	iNKT	0.106077	0.136774	0.044715	0.063343	0.0035
	Treg	5.700256	1.394416	6.43209	2.0921	0.0471
	Tresp	59.58974	6.915383	58.87761	9.916268	0.3774
B cells	CD19+	9.916923	2.700009	10.9091	5.710798	0.2173
	Bm1	11.02256	4.493864	6.626716	5.256401	0.0004
	Bm2	64.13846	8.804473	63.60896	14.99722	0.4416
	Bm2 (Transitional)	3.172564	2.077853	6.268955	7.495644	0.0163
	Bm3-Bm4	0.734615	0.455983	1.208955	1.109305	0.0163
	Early Bm5	13.14077	5.14155	14.79791	9.811632	0.2207
	Late Bm5	7.391282	3.133523	6.949507	5.74571	0.3725
	CD19+ Naïve	63.03846	10.78782	69.12985	17.32764	0.0461
	CD19+ Switched memory	15.01872	6.407468	13.14313	9.703069	0.2087
	CD19+ Unswitched memory	15.75821	5.842608	7.980746	4.605099	0.0005
Monocytes	CD14+	10.16026	3.432555	14.07358	6.892336	0.0027
	Classical	86.54615	6.71745	87.34179	6.187675	0.3293
	Intermediate	2.946667	1.625606	3.45791	2.099741	0.1581
	non-classical	6.195385	4.514826	5.158358	4.178138	0.1813
PDC's	PDC's	0.371795	0.138524	0.277597	0.15336	0.0035

Summary of the JSLE immune cell signature
Table displaying the mean expression frequencies and standard deviations of the immune cell types in 39 HCs and 67 JSLE patients. Immune cell subsets selected as the JSLE-immune cell signature - identified and validated from the BRF model, logistic regression and sPLS-DA (Appendix p. 11) shown in green. P values from unpaired t-tests followed by 5% false discovery rate adjustment for multiple comparisons (Benjamini, Krieger and Yekutieli)[5].

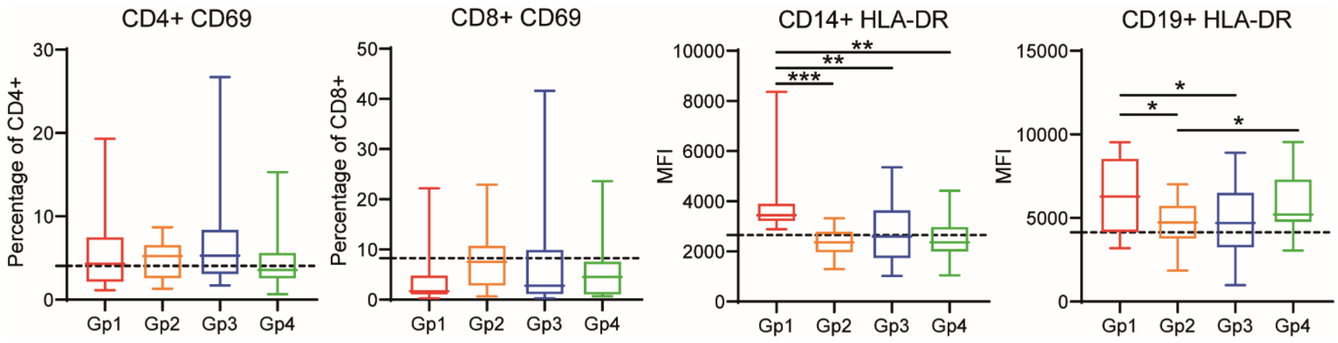

Comparison of activation markers in T-cells, B-cells and monocytes PBMC's from HCs (n=39) and JSLE patients (n=67) were stained ex-vivo to evaluate the surface expression of CD69 in CD4+ and CD8+ T-cells and HLA-DR in CD19+ B-cells and CD14+ monocytes by flow cytometry across k means clustered JSLE groups. Mean+SE, one-way ANOVA, Tukey's multiple comparisons test, *=p<0.05, **=p<0.01, **=p<0.001. Dotted lines represent HC average. Refer to Figure 5E.

Clinical comparison between k-means clustered groups

	Group 1	Group 2	Group 3	Group 4	p-value
Total number	10	21	21	15	-
Female:Male	9:1	15:6	19:2	11:4	0.3194
Median age	20.5 (18-21)	18 (16-21.5)	20 (17-22)	20 (17-21)	*0.5407
Body Mass Index (BMI), median (IQR)	22.3 (20.1-25.7)	21.5 (20.8-26.7)	22.4 (20.1-26.8)	22.8 (19.4-30.5)	*0.7471
**Ethnicity, number (%):**					
White	3 (30%)	8 (38%)	3 (14%)	6 (40%)	0.2782
Asian	7 (70%)	9 (43%)	2 (10%)	6 (40%)	0.0957
Black	0 (0%)	5 (24%)	7 (33%)	5 (33%)	0.2008
Other/unknown	1 (10)	1 (5%)	2 (10%)	2(13%)	0.8429
**Disease characteristics**					
Age of diagnosis, mean (range)	12.5 (10.5-15)	13 (10-14)	13 (10.5-16.5)	11 (9-14)	*0.6600
Disease duration, mean (range) (years)	6.5 (5.8-10)	5 (2.5-9)	7 (3-10)	9 (5-11)	*0.6946
SLEDAI, median (IQR)	3.36 (1.25-4.98)	7.48 (3.8-9.04)	4.80 (2.84-8.02)	4.94 (3.69-14.2)	*0.1944
SLICC, mean (range)	0.1000 (0.32)	0.04762 (0.22)	0.3333 (0.66)	0.06667 (0.26)	0.1330
LLDAS (% in LLDAS)	70.0	85.7	66.7	66.7	0.4739
**Serology [median (IQR)]:**					
dsDNA (IU/mL) (NR=<50)	53.5 (12-335)	6 (2.5-117.5)	46.5 (4.25-384.5)	9 (2-47)	*0.3938
dsDNA (% outside NR)	50.0	33.3	38.1	20.0	0.4580
CRP (mg/L) (NR<5)	0.8 (0.65-1.35)	0.6 (0.6-1.55)	1.65 (0.6-5.2)	1.5 (0.65-4.25)	*0.0948
CRP (% outside NR)	10.0	9.5	23.8	13.3	0.5706
C3 (g/L) (NR=0.9-1.8) (mean (range))	0.71 (0.58-0.94)	1.09 (0.93-1.23)	0.84 (0.64-1.16)	1.11 (0.98-1.22)	*0.0011 (Gp2 vs Gp3, Gp2 vs Gp1, Gp3 vs Gp4, Gp4 vs Gp1)
C3 (% outside NR)	60.0	42.9	52.4	33.3	0.5355
LC (10^/L) (NR=1.3-3.5)	1.2 (1.16-1.42)	1.37 (1.1-1.99)	1.13 (0.75-1.65)	1.45 (1.1-2.23)	*0.0844
LC (% outside NR)	70.0	42.9	61.9	33.3	0.1814
NC (10^9/L) (NR=2.0 - 7.5)	2.58 (1.87-3.65)	3.01 (2.1-4.27)	3.87 (2.33-4.82)	2.68 (2.58-5.78)	*0.7400
NC (% outside NR)	50.0	23.8	19.0	26.7	0.3237
UP:C ratio (mg/mmol) (NR=0-13)	7.5 (6-20)	9 (6-13)	8 (2-14.5)	8 (6-12)	*0.3474
UP:C (% outside NR)	30.0	19.0	28.6	20.0	0.8364
Haemoglobin (g/L) ((NR=115–155)	130 (112-136.3)	128 (114-147)	119 (105-125)	120 (107-133)	*0.1244
Haemoglobin (% outside NR)	30.0	38.1	33.3	46.7	0.8164
Platelet count (10^9/L) ((NR=150–400)	224 (184.8-277.5)	265 (205-338)	292 (228.5-317)	337 (268-393)	*0.0863
Platelet count (% outside NR)	0.0	9.5	9.5	20.0	0.4457
Antinuclear antibodies (ANA) (% positive)	80.0	66.7	95.2	86.7	0.1064
Extractable Nuclear Antigens (ENA) (% positive)	80.0	52.4	57.1	66.7	0.4734
**Clinical lipids [median (IQR)]:**					
Cholesterol (NR<5mmol/L)	4.5 (3.4-5.1)	3.9 (3.4-4.2)	4.2 (3.8-4.5)	3.7 (3.3-4.2)	*0.0938
Triglycerides (NR<3mmol/L)	1.3 (0.8-2.1)	0.8 (0.5-0.9)	0.9 (0.6-1.3)	0.8 (0.5-1.2)	*0.0990
HDL-C (NR>1mmol/L)	1.4 (1.1-1.9)	1.5 (1.2-1.7)	1.3 (1-1.5)	1.6 (1.2-1.8)	*0.2073
LDL-C (NR<3mmol/L)	2.2 (1.2-3.3)	1.8 (1.6-2.3)	2.4 (1-1.6)	2 (1.3-2.1)	*0.1341
**Current treatment [n (%)]:**					
Hydroxychloroquine	9 (90%)	19 (90.5%)	20 (95.2%)	13 (86.7%)	0.8429
Mycophenolate mofetil	6 (60%)	12 (57.1%)	4 (19%)	4 (26.7%)	0.0262
Prednisolone	4 (40%)	11 (52.4%)	9 (42.9%)	8 (53.3%)	0.8470
Vitamin D	1 (10%)	4 (19%)	4 (19%)	4 (26.7%)	0.7831
Methotrexate	0 (0%)	1 (4.8%)	2 (9.5%)	3 (20%)	0.2971
Azathioprine	2 (20%)	5 (23.8)	5 (23.8%)	3 (20%)	0.9879
**Comorbidity [n (%)]:**					
Antiphospholipid syndrome	1 (10%)	1 (4.8%)	1 (4.8%)	0 (0%)	0.6999
Juvenile dermatomyositis	0 (0%)	0 (0%)	2 (9.5%)	2 (14.3%)	0.2737
Polyarticular juvenile idiopathic arthritis	0 (0%)	2 (9.5%)	2 (9.5%)	0 (0%)	0.4799
Sjogren's syndrome	0 (0%)	2 (9.5%)	1 (4.8%)	0 (0%)	0.4888

Clinical comparison between k-means clustered groups. For patients the SLEDAI score was calculated. Other common clinical measures of disease are shown as well as treatments. Patients with rituximab treatment were not included in the cohort. Chi-square test or *one-way ANOVA was used. Abbreviations: NR: Normal ranges, SLEDAI: Systemic Lupus Erythematosus Disease Activity Index, dsDNA: Anti-double-stranded-DNA antibodies, C3: Complement component 3, LC: Lymphocyte count, NC: Neutrophil count, UP:C: Urine protein:Creatinine ratio, CRP: C-reactive Protein. SLICC: Systemic Lupus International Collaborating Clinics, LLDAS: Lupus Low Disease Activity State.

Longitudinal treatment analysis across k-means clustered JSLE groups

	Group 1	Group 2	Group 3	Group 4	p-value
**Total number**	10	21	21	15	-
**Treatment (mg), mean (SD)**					
**Average hydroxychloroquine**	250 (112)	300 (122)	311 (93)	343 (83)	0.1960
**% visits on hydroxychloroquine**	87 (31)	89 (29)	91 (24)	98 (3.3)	0.6224
**Average prednisolone**	6.9 (5.3)	4.8 (6.1)	4.3 (4.8)	4.6 (5.1)	0.6502
**% visits on prednisolone**	63 (39)	41 (42)	47 (47)	50 (45)	0.6192
**Average mycophenolate mofetil, g**	3.3 (5.9)	0.72 (0.7)	0.70 (0.94)	1.2 (1.0)	Gp1vsGp2: 0.0059, Gp1vsGp3: 0.0056, Gp1vsGp4: 0.0371
**% visits on mycophenolate mofetil**	63 (44.84)	44 (44.09)	28 (42.62)	53 (40.34)	Gp1vsGp3: 0.0361
**Average methotrexate**	1.4 (4.4)	0.36 (1.6)	2.3 (6.1)	3.5 (6.9)	0.3202
**% visits on methotrexate**	7.8 (25)	0.30 (1.4)	9.8 (30)	15 (30)	0.3423
**Average azathioprine**	53 (98)	43 (79)	34 (57)	52 (81)	0.8903
**% visits on azathioprine**	14 (32)	21 (38)	26 (43)	15 (30)	0.7951
**Average cumulative cyclophosphamide**	0.82 (1.4)	1.4 (2.7)	1.6 (2.7)	0.21 (0.8)	0.2941
**% visits on cyclophosphamide**	38 (47)	30 (44)	36 (48)	7.1 (27)	0.2081
**% visits on IVIG**	0 (0)	0.76 (2.4)	7.6 (20)	7.1 (27)	0.4195
**% visits on rituximab/ofatumumab**	5.7 (6.2)	2.4 (4.7)	4.9 (8.6)	2.7 (5.3)	0.4317
**Organ involvement developed, n (%)**					
**Renal**	5 (50%)	2 (9.5%)	4 (19%)	4 (26.7%)	*0.0828
**Central nervous system**	1 (10%)	2 (9.5%)	1 (4.8%)	0 (0%)	*0.6218
**Cardiovascular**	0 (0%)	0 (0%)	3 (14.3%)	1 (4.8%)	*0.2063
**Musculoskeletal**	2 (20%)	5 (23.8%)	5 (23.8%)	7 (33.3%)	*0.3552
**Haematological**	4 (40%)	9 (42.9%)	11 (52.4%)	5 (23.8%)	*0.7153
**Gastrointestinal**	0 (0%)	0 (9%)	1 (4.8%)	0 (0%)	*0.5273
**Skin**	8 (80%)	14 (66.7%)	12 (57.1%)	10 (47.6%)	*0.6585

Longitudinal treatment and organ involvement analysis across k-means clustered JSLE groups. Longitudinal analysis, across k-means clustered JSLE groups, of patient disease specific treatment and organ involvement data between 3-7 years of follow up (mean follow-up per patient=4.9 years, mean number of visits per patient=17.1). Organ involvement was assessed based on clinician opinion who completed the SLEDAI score for every patient at every visit, based on a combination of: clinical picture routine blood test results, biopsy results (as appropriate (eg renal, skin). Data is reported as mean (SD) for average dose in mg (except for MMF which is g) and/or percentage of visits on the treatment. One-way ANOVA or *Chi-square test was used. Significant p values are displayed in red. IVIG: Intravenous Immunoglobulin. Refer to Figure 5H

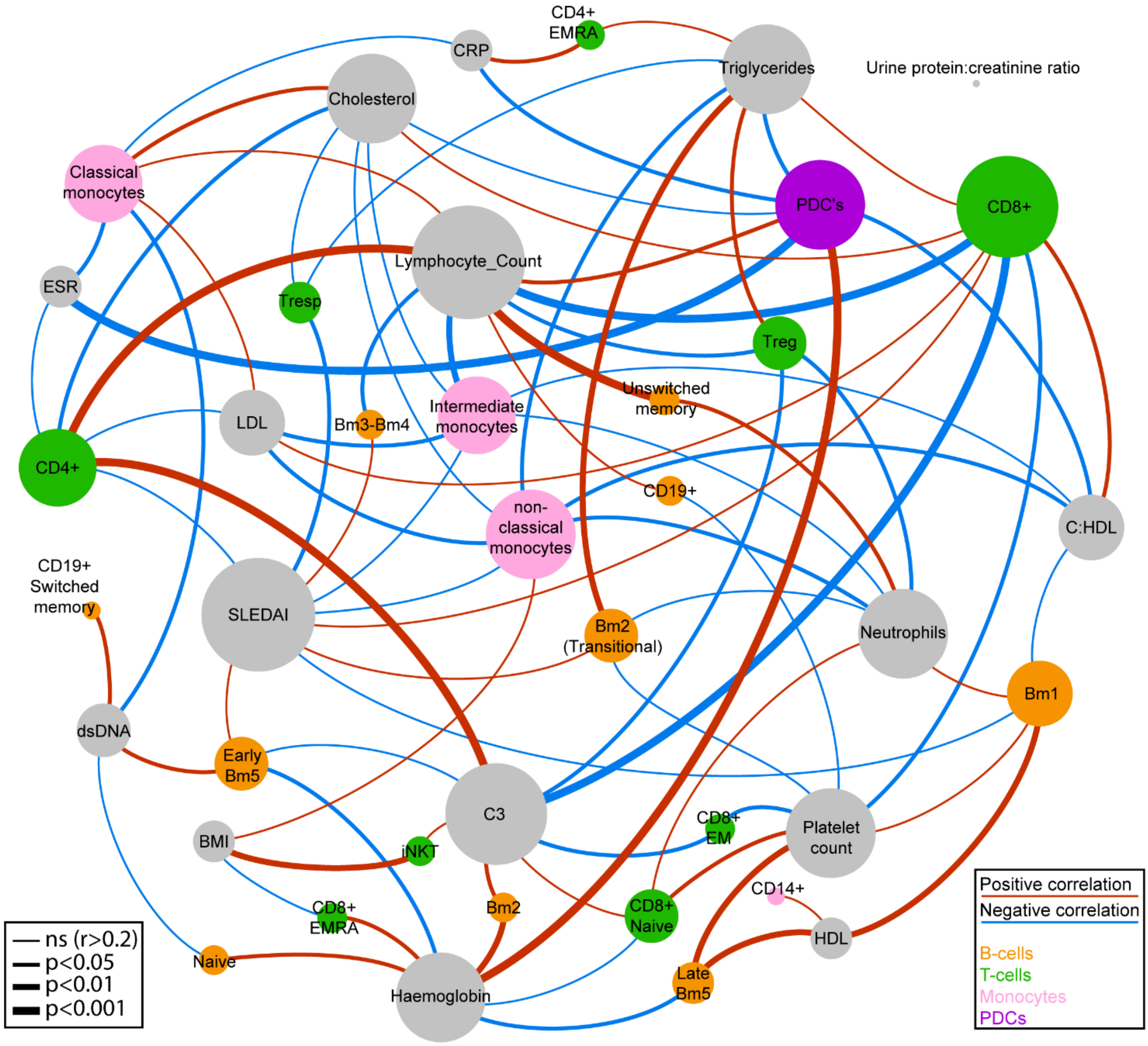

Network analysis identifies associations between immunological and clinical features JSLE. Correlations between immune cell frequency and JSLE clinical characteristics. Pearson correlation coefficients based on univariate logistic regression are represented as connecting lines (edges) between the clinical characteristic nodes and immune cell frequency nodes. The width of the connecting edges represent the significance of the correlation. Only correlations with an absolute r value of 0.2 and above are shown. P values and r values are displayed in Appendix p. 17. Size of the circles (nodes) are proportional to the total number of connections with other nodes. Red line=positive correlation and blue line=negative correlation. Node colour was grouped according to immune cell type (T-cells: green, B-cells: orange, monocytes: pink, PDC's: purple) and clinical characteristic (grey). The graph was generated using the Force Atlas layout in Gephi 0.9.2.

	ESR	dsDNA	C3	Lymphocyte Count	CRP	Cholesterol	Triglycerides	HDL	LDL	C:HDL	Neutrophils	UP:C	Haemoglobin	Platelet count	BMI	SLEDAI
CD4+	-0.23 (0.073)	NA	0.48 (0.0001)	0.44 (0.0004)	NA	-0.27 (0.0332)	NA	NA	-0.24 (0.0646)	NA	NA	NA	NA	NA	NA	-0.23 (0.0739)
CD4+ CM	NA	NA	NA	NA	NA	NA	NA	NA	NA	NA	NA	NA	NA	NA	NA	NA
CD4+ EM	NA	NA	NA	NA	NA	NA	NA	NA	NA	NA	NA	NA	NA	NA	NA	NA
CD4+ EMRA	NA	NA	NA	NA	0.21 (0.0144)	NA	0.24 (0.0588)	NA	NA	NA	NA	NA	NA	NA	NA	NA
CD4+ Naive	NA	NA	NA	NA	NA	NA	NA	NA	NA	NA	NA	NA	NA	NA	NA	NA
CD8+	NA	NA	-0.50 (0.0001)	-0.41 (0.0009)	NA	0.24 (0.0586)	0.23 (0.0692)	NA	0.22 (0.0897)	0.27 (0.0370)	NA	NA	NA	-0.28 (0.0288)	NA	0.22 (0.0765)
CD8+ CM	NA	NA	NA	NA	NA	NA	NA	NA	NA	NA	NA	NA	NA	NA	NA	NA
CD8+ EM	NA	NA	-0.30 (0.0156)	NA	NA	NA	NA	NA	NA	NA	NA	NA	NA	-0.31 (0.0147)	NA	NA
CD8+ EMRA	NA	NA	NA	NA	NA	NA	NA	NA	NA	NA	NA	NA	0.28 (0.0256)	NA	-0.23 (0.0762)	NA
CD8+ Naive	NA	NA	0.20 (0.1141)	NA	NA	NA	NA	NA	NA	NA	0.23 (0.0646)	NA	-0.20 (0.1090)	0.28 (0.0246)	NA	NA
iNKT	NA	NA	0.23 (0.0668)	NA	NA	NA	NA	NA	NA	NA	NA	NA	NA	NA	0.33 (0.0074)	NA
Treg	NA	NA	-0.28 (0.0271)	-0.25 (0.0495)	NA	NA	0.27 (0.0363)	NA	NA	NA	-0.29 (0.0234)	NA	NA	NA	NA	NA
Tresp	NA	NA	NA	NA	NA	-0.23 (0.0735)	-0.23 (0.0693)	NA	NA	NA	NA	NA	NA	NA	NA	-0.26 (0.0383)
CD19+	NA	NA	NA	0.23 (0.0734)	NA	NA	NA	NA	NA	NA	NA	NA	NA	-0.24 (0.0624)	NA	NA
Bm1	NA	NA	NA	NA	NA	NA	NA	0.38 (0.0022)	NA	-0.23 (0.0661)	0.21 (0.1048)	NA	NA	0.22 (0.0836)	NA	-0.23 (0.0697)
Bm2	NA	NA	0.25 (0.0447)	NA	NA	NA	NA	NA	NA	NA	NA	NA	0.39 (0.0015)	NA	NA	NA
Bm2 (Transitional)	NA	NA	NA	NA	NA	NA	0.35 (0.0050)	NA	NA	NA	-0.22 (0.0853)	NA	NA	-0.22 (0.0833)	NA	0.21 (0.1024)
Bm3-Bm4	NA	NA	NA	-0.26 (0.0402)	NA	NA	NA	NA	NA	NA	NA	NA	NA	NA	NA	0.21 (0.1042)
Early Bm5	NA	0.29 (0.0200)	-0.23 (0.0758)	NA	NA	NA	NA	NA	NA	NA	NA	NA	-0.30 (0.0186)	NA	NA	0.23 (0.0712)
Late Bm5	NA	NA	NA	NA	NA	NA	NA	0.36 (0.0059)	NA	NA	NA	NA	-0.31 (0.0126)	0.33 (0.0077)	NA	NA
CD19+ Naive	NA	-0.20 (0.1101)	NA	NA	NA	NA	NA	NA	NA	NA	NA	NA	0.28 (0.0285)	NA	NA	NA
CD19+ Switched memory	NA	0.30 (0.0168)	NA	NA	NA	NA	NA	NA	NA	NA	NA	NA	NA	NA	NA	NA
CD19+ Unswitched memory	NA	NA	NA	0.41 (0.0007)	NA	NA	NA	NA	NA	NA	0.26 (0.0413)	NA	NA	NA	NA	NA
CD14+	NA	NA	NA	NA	NA	NA	NA	0.21 (0.0996)	NA	NA	NA	NA	NA	NA	NA	NA
Classical	-0.28 (0.0247)	-0.30 (0.0182)	NA	0.22 (0.0834)	-0.24 (0.0560)	0.26 (0.0377)	NA	NA	0.22 (0.0807)	NA	NA	NA	NA	NA	NA	NA
Intermediate	NA	NA	NA	-0.33 (0.0084)	NA	-0.22 (0.0809)	NA	NA	-0.29 (0.0220)	-0.23 (0.0675)	-0.24 (0.0589)	NA	NA	NA	NA	-0.23 (0.0668)
non-classical	NA	NA	NA	NA	NA	-0.30 (0.0198)	-0.26 (0.0450)	NA	-0.25 (0.0462)	-0.30 (0.0178)	-0.28 (0.0252)	NA	NA	NA	0.23 (0.0662)	-0.20 (0.1083)
PDC's	-0.47 (0.0001)	NA	NA	0.30 (0.0158)	-0.26 (0.0388)	-0.24 (0.0645)	-0.32 (0.0105)	NA	NA	-0.29 (0.0243)	NA	NA	0.41 (0.0007)	NA	NA	NA

Network analysis comparing immunophenotype to clinical measures in JSLE. Correlation analysis was performed using R software on 28 immune cell subsets from 67 JSLE patients. Pearson correlation coefficients based on univariate logistic regression are presented between the clinical characteristics and immune cell frequencies. Only correlations with an absolute r value of 0.2 and above are displayed, P values displayed in brackets and are shown in red where $p<0.05$. NA=values below r=<0.2. See Appendix p. 16

References

1. Hochberg MC. Updating the American college of rheumatology revised criteria for the classification of systemic lupus erythematosus. *Arthritis & Rheumatism* 1997; **40**(9): 1725-.
2. Petri M, Orbai A-M, Alarcón GS, et al. Derivation and validation of the Systemic Lupus International Collaborating Clinics classification criteria for systemic lupus erythematosus. *Arthritis & Rheumatism* 2012; **64**(8): 2677-86.
3. Yee C-S, Farewell VT, Isenberg DA, et al. The use of Systemic Lupus Erythematosus Disease Activity Index-2000 to define active disease and minimal clinically meaningful change based on data from a large cohort of systemic lupus erythematosus patients. *Rheumatology* 2010; **50**(5): 982-8.
4. Franklyn K, Lau CS, Navarra SV, et al. Definition and initial validation of a Lupus Low Disease Activity State (LLDAS). *Annals of the Rheumatic Diseases* 2016; **75**(9): 1615-21.
5. Benjamini Y, Krieger AM, Yekutieli D. Adaptive linear step-up procedures that control the false discovery rate. *Biometrika* 2006; **93**(3): 491-507.
6. R Core Team. R: A language and environment for statistical computing. Available online at https://www.R-project.org/. 2018.
7. Diedenhofen B, Musch J. cocor: A Comprehensive Solution for the Statistical Comparison of Correlations. Figshare; 2015.
8. Warnes, MGR., Bolker, B., Bonebakker, L., Gentleman, R., Package gplots. 2016.
9. Liaw, A. & Wiener, M. Classification and regression by randomForest. R News 2, 18-22 (2002).
10. Ho TK. Random decision forests. Proceedings of the Third International Conference on Document Analysis and Recognition (Volume 1) - Volume 1: IEEE Computer Society; 1995. p. 278.
11. Robin X, Turck N, Hainard A, et al. pROC: an open-source package for R and S+ to analyze and compare ROC curves. *BMC Bioinformatics* 2011; **12**(1): 77.
12. Rohart F, Gautier B, Singh A, Lê Cao K-A. mixOmics: An R package for ‘omics feature selection and multiple data integration. *PLOS Computational Biology* 2017; **13**(11): e1005752.
13. Kuhn, M The caret package. R Foundation for Statistical Computing, Vienna. URL https://cran.r-project.org/package=caret.2012
14. Wilkinson L. ggplot2: Elegant Graphics for Data Analysis by WICKHAM, H. *Biometrics* 2011; **67**(2): 678-9.
15. R Core team (2018). R: A language and environment for statistical computing. R Foundation for Statistical Computing, Vienna, Austria. https://www.R-project.org/.
16. Bastian M, Heymann S, Jacomy M. Gephi: An Open Source Software for Exploring and Manipulating Networks; 2009.
